# Dysfunction of the noradrenergic system drives inflammation, α-synucleinopathy, and neuronal loss in mouse colon

**DOI:** 10.3389/fimmu.2023.1083513

**Published:** 2023-02-10

**Authors:** Sheng Song, Dezhen Tu, Chengbo Meng, Jie Liu, Belinda Wilson, Qingshan Wang, Yen-Yu Ian Shih, Hui-Ming Gao, Jau-Shyong Hong

**Affiliations:** ^1^ Neuropharmacology Section, Neurobiology Laboratory, National Institute of Environmental Health Sciences, National Institutes of Health, Research Triangle Park, NC, United States; ^2^ Biomedical Research Imaging Center, University of North Caroline at Chapel Hill, Chapel Hill, NC, United States; ^3^ Ministry of Education (MOE) Key Laboratory of Model Animal for Disease Study, Institute for Brain Sciences, Jiangsu Key Laboratory of Molecular Medicine, Model Animal Research Center, School of medicine, Nanjing University, Nanjing, China; ^4^ In Vivo Neurobiology Group, Neurobiology Laboratory, National Institute of Environmental Health Sciences, National Institutes of Health, Research Triangle Park, NC, United States; ^5^ National-Local Joint Engineering Research Center for Drug-Research and Development (R & D) of Neurodegenerative Diseases, Dalian Medical University, Dalian, China

**Keywords:** α-synuclein, noradrenergic system, gut-brain axis, inflammation, Parkinson’s disease

## Abstract

Clinical and pathological evidence revealed that α-synuclein (α-syn) pathology seen in PD patients starts in the gut and spreads *via* anatomically connected structures from the gut to the brain. Our previous study demonstrated that depletion of central norepinephrine (NE) disrupted brain immune homeostasis, producing a spatiotemporal order of neurodegeneration in the mouse brain. The purpose of this study was 1) to determine the role of peripheral noradrenergic system in the maintenance of gut immune homeostasis and in the pathogenesis of PD and 2) to investigate whether NE-depletion induced PD-like α-syn pathological changes starts from the gut. For these purposes, we investigated time-dependent changes of α-synucleinopathy and neuronal loss in the gut following a single injection of DSP-4 (a selective noradrenergic neurotoxin) to A53T-SNCA (human mutant α-syn) over-expression mice. We found DPS-4 significantly reduced the tissue level of NE and increased immune activities in gut, characterized by increased number of phagocytes and proinflammatory gene expression. Furthermore, a rapid-onset of α-syn pathology was observed in enteric neurons after 2 weeks and delayed dopaminergic neurodegeneration in the substantia nigra was detected after 3-5 months, associated with the appearance of constipation and impaired motor function, respectively. The increased α-syn pathology was only observed in large, but not in the small, intestine, which is similar to what was observed in PD patients. Mechanistic studies reveal that DSP-4-elicited upregulation of NADPH oxidase (NOX2) initially occurred only in immune cells during the acute intestinal inflammation stage, and then spread to enteric neurons and mucosal epithelial cells during the chronic inflammation stage. The upregulation of neuronal NOX2 correlated well with the extent of α-syn aggregation and subsequent enteric neuronal loss, suggesting that NOX2-generated reactive oxygen species play a key role in α-synucleinopathy. Moreover, inhibiting NOX2 by diphenyleneiodonium or restoring NE function by salmeterol (a β2-receptor agonist) significantly attenuated colon inflammation, α-syn aggregation/propagation, and enteric neurodegeneration in the colon and ameliorated subsequent behavioral deficits. Taken together, our model of PD shows a progressive pattern of pathological changes from the gut to the brain and suggests a potential role of the noradrenergic dysfunction in the pathogenesis of PD.

## 1 Introduction

Parkinson’s disease (PD) is a neurological disease clinically defined by degeneration of dopaminergic neurons in substantia nigra. One of the cardinal characteristics of PD is its progressive nature. However, the precise pathological mechanisms underlying the disease progression remain unclear. Currently, treatment options for PD are limited to symptoms relief, predominantly in the form of dopaminergic medications and deep brain stimulation (1–3). Whilst, the disease-modifying strategies aimed to stop PD progression are still lacking. Recent findings in understanding PD progression have greatly facilitated both basic and clinical research. Based on the progression of Lewy pathology in the brain, Braak’s group has proposed a neuropathological temporal staging scheme in a caudal-rostral pattern for PD (4). In PD patients, neuronal loss starts from the lower brain (including raphe nucleus and Locus coeruleus (LC)) and olfactory bulb, then gradually affects the higher centers of the brain (5). Further studies uncovered that persistent peripheral inflammation and gut dysfunction occurs years before PD patients show movement disorders (6–10). The proposed route of disease progression originating from the gut and spread to the brain *via* anatomically connected structures fits well with the progression of PD symptoms (11, 12). Before movement symptoms occur, premotor symptoms, such as constipation (an indicator of gut dysfunction), hyposmia, sleep disorder and other symptoms related to autonomic dysfunction, are often observed in patients with PD (4, 13).

The role of gut dysfunction in PD has been becoming an intense research topic. A stage-development of PD model showed that α-synuclein (α-syn) aggregates were initially found in the intestine, progressed to the medulla oblongata, and then to the substantia nigra pars compacta (SNpc) (4, 14, 15). Pathological α-syn fibrils locally injected into the gut was found to spread to the brain as evidenced by appearance of S129 phosphorylated α-syn first in the dorsal motor nucleus, then in caudal portions of the hindbrain (including the LC), and much later in the basolateral amygdala, dorsal raphe nucleus, and the SNpc (16); Similar findings have also been demonstrated by inoculation of α-syn fibrils in aged mice, which resulted in progression of α-syn pathology to the midbrain and subsequent motor defects (17). The microtubule-associated transport is involved in the translocation of aggregated α-synuclein in neurons, which enabled different α-syn forms (monomeric, oligomeric and fibrillar) to propagate from the gut to the brain (18). Moreover, previous studies have proved that PD is linked with gut microbial dysbiosis in both human beings and animals (19–22), the gut microbiome also produces functional amyloids and promotes α-syn aggregation from the gut to the brain in a prion-like fashion (23).

We have recently reported that selective norepinephrine (NE)-depleting toxin N-(2-chloroethyl)-N-ethyl-2-bromobenzylamine (DSP-4) produced a time-dependent neurodegenerative pattern similar to that described by Braak et al. (24). Noradrenergic dysfunction produced by DSP-4 also enhanced LPS-induced dopaminergic neuron loss in the SNpc (25), and accelerated LPS-elicited inflammation-related neurodegeneration in an ascending sequential manner, together with behavioral dysfunctions (26). Furthermore, changes in microbiota composition in the colon were observed after DSP-4 injection (27). However, little is known about whether inflammation occurs in the gut in mice with NE-depletion in the enteric nervous system. We speculated that NE dysfunction may produce chronic intestinal inflammation by disrupting gut immune homeostasis, which in turn affects the composition in colon microbiota. Using mutant human α-syn-overexpressing mice injected with DSP-4, we studied 1) temporal relationship among inflammation, α-synucleinopathy and neuronal loss in colon and 2) effects of elimination of colon inflammation by anti-inflammatory agents, such as long-lasting β2-adrenoreceptor (β2-AR) agonist salmeterol or NADPH oxidase (NOX2) inhibitor dipheyleneiodonim (DPI), on α-synucleinopathy and neuronal loss in colon as well as the defecatory dysfunction. The results showed that profound inflammation occurred within 1-2 weeks after DSP-4 exposure and became permanent in the colon, but not in the small intestines. Colon inflammation was followed by increases in α-synucleinopathy and neuronal loss in colon. Taken together, this report provides the first evidence indicating loss of gut norepinephrine contributes to the formation of colon inflammation, which is likely leading to α-syn/gut pathology.

## 2 Materials and methods

### 2.1 Animals and treatment

Male homozygous transgenic mice over-expressing human A53T mutant α-syn (B6.C3-Tg [Prnp-SNCA*A53T] 83 Vle/J) and male CX3CR-1GFP mice (B6.129P2(Cg)-Cx3cr1^tm1Litt^/J) were obtained from the Jackson Laboratory (Bar Harbor, ME). Housing and breeding of animals were performed humanely with regard to the alleviation of suffering following the National Institutes of Health’s Guide for the Care and Use of Laboratory Animals (Institute of Laboratory Animal Resources, 2011). A single i.p. injection of DSP-4 (50 mg/kg; Sigma-Aldrich, St. Louis, MO) or vehicle (saline, 5 ml/kg, i.p.) was administered to 8-week-old male SNCA mice to deplete NE in the peripheral. Two days later, DSP-4-treated SNCA mice received either salmeterol at 10 μg/kg/day or DPI at 10 ng/kg/day for 4 weeks *via* an Alzet osmotic pump implanted subcutaneously. The dosage of salmeterol (28) or DPI (24) was selected based on our previous studies.

Mice were housed on a 12 h:12 h light: dark schedule with lights on at 7 AM. All behavioral testing was conducted during the light phase of the light/dark cycle. All the experimental mice are transferred to the behavior testing room 30 min prior to beginning the first trial to habituate to the condition of the behavior testing room. Testing was conducted under fluorescent laboratory lighting (180-205 lx for the Morris water maze; 320-340 lx for elevated plus maze and 3-chamber social choice test). All procedures were approved by the National Institutes of Environmental Health Sciences Animal Care and Use Committee.

### 2.2 Immunohistochemistry

Mice were euthanized using fatal plus at the desired time points after injection. Under deep anesthesia, mice were rapidly transcardially perfused with 15 ml of ice-cold phosphate-buffered saline solution (PBS) containing heparin for approximately 2 minutes, followed by perfusion with ice-cold 4% paraformaldehyde (PFA; 25 ml at 5 ml/min). The intestines were excised from each mouse and were cut longitudinally, and luminal contents were washed out using PBS. After unfolding on filter papers, the intestines were prepared in a Swiss roll (29) and embedded in paraffin.

Paraffin-embedded intestines were sectioned in 8 μm thick and processed for immune-staining as described previously (26). Briefly, after deparaffinization and rehydration, the sections were preincubated for an hour in 10% normal goat serum blocking solution (Vector laboratories, S-1000-20), 1% bovine serum albumin (BSA) und 0.4% Triton X-100 in 0.1 M PBS. This was followed by an incubation with the primary antibodies in antibody diluent (Dako, S080983-2) overnight at 4°C. The second antibody was a biotinylated secondary goat-anti-rabbit/mouse-antibody (Vector laboratories, 1:100; rabbit, BA-5000-1.5; mouse, BA-9200-1.5) in antibody diluent, which was applied for 1 hour at room temperature. We used the following primary antibodies for immunohistochemistry: antibodies against α-syn phosphorylated on Ser129 (p-α-syn, rabbit, ab51253, 1:2000, Abcam), CD45 (rabbit, ab10558, 1:1000, Abcam), gp91^phox^ (mouse, 1:500, BD Biosciences, 611414), Muc2 (mouse, ab97386, 1:3000, Abcam). After an additional 1 h incubation in an avidin/biotin solution, immunostaining was visualized by using 3,3’-diaminobenzidine (DAB) substrate kit (Vector laboratories, SK-4100). The sections were further counterstained with hematoxylin for visualizing nuclei according to the standard protocol.

### 2.3 Tissue clearing

We performed passive CLARITY tissue clearing (PACT) as described by Yang et al. (30). Large intestines were fixed in 4% PFA for 16 hours, followed by 3 rinses in PBS. Tissues were then incubated in hydrogel monomer solution AP40 (4% v/v acrylamide and 0.25% w/v VA-044 in PBS) for 72 hours at 4°C, protected from light. Oxygen was then removed from the samples using a chamber connected to vacuum and nitrogen, followed by incubation at 37°C for 3 hours to initiate tissue-hydrogel hybridization. The hydrogel was removed from the tissues *via* 3X washes with PBS, and tissues were subsequently incubated in 8% SDS prepared in PBS for 7 days at 37°C with shaking, and the SDS solution was replaced twice during incubation. The tissues were then washed 5 times in PBS (one hour each time) and blocked in 5% NDS prepared in PBS/Triton X-100 with 0.01% of sodium azide. The samples were then incubated in primary antibody prepared in antibody diluent (2% v/v NDS, 0.01% w/v sodium azide in PBST) for 6 days at room temperature with constant rotation, followed by 5X one-hour washes in 0.1% v/v Triton in PBS (PBS-T). Secondary antibody was similarly prepared in antibody diluent and incubated for another 6 days at room temperature with constant rotation and protected from light, replacing antibody halfway through incubation. Finally, the samples were washed an additional 5 times (one hour each time) in PBS-T and incubated in Refractive Index Matching Solution (80% w/v Histodenz (catalog no. D2158, Sigma-Aldrich) prepared in 0.02M phosphate buffer, pH7.5 with 0.1% Tween-20 and 0.01% sodium azide, refractive index = 1.46) for 1-3 days, and samples were mounted in fresh Reflective Index Mounting Solution using a 1 mm deep iSpacer (SUNjin Lab).

### 2.4 Confocal image collection and processing

Tile scan images of tissue cleared by PACT were collected on an LSM 880 confocal microscope using an EC Plan-Neofluar 10x/0.3 M27 objective (Carl Zeiss, Thornwood, NY) (31). Due to the thickness of the samples, the Auto Z Brightness Correction was used for both the 488 nm laser line from an Argon laser (2–20% power range) and 561 nm laser line from a DPSS laser (2–20% power range). The pinhole was set to yield an optical z-thickness of 14 μm, and a z-stack was collected at 5 μm interval between images. z-stacks were viewed with Imaris Software (Bitplane, Concord MA) for three-dimensional rendering of the entire region and surface rendering of individual cells.

The paraffin-embedded sections were processed and immune-blocked with 4% goat serum in 0.25% triton/PBS for 2 hours and then incubated with antibodies against HuC/D (ab184267, rabbit, 1:500, Abcam, Cambridge, MA), 3-nitrotyrosine (3-NT, mouse, 1:200, Abcam, Cambridge, MA), Muc2 (mouse, ab97386, 1:3000, Abcam), Chromogranin A (CgA, rabbit, ab45179, 1:2000, Abcam), ionized calcium binding adaptor molecule-1 (Iba-1; rabbit, 1:2000, Wako Chemicals, Richmond, VA) or gp91^phox^ (mouse, 1:500, BD Biosciences, 611414) overnight at 4°C. On the second day, the sections were washed with 1% BSA in 0.25% triton/PBS before incubation with anti-TH antibody (mouse: MAB318, 1:2000; rabbit: AB152, 1:2000; EMD Millipore, Temecula, CA) overnight at 4°C. The double/triple-label immunofluorescence images after staining with Alexa-488 (green), Alexa-594 (red), and/or Alex-647 conjugated secondary antibodies (1:1,000) were taken under the confocal microscope to visualize the immunoreactive cells. Densitometry was performed using ImageJ.

### 2.5 Cell counts

The count of HuC/D-positive cells in a segment of cleared tissue from the same location of each large intestine was conducted by Imaris software using the ‘Spots’ feature. Briefly, confocal image stacks were reconstructed and visualized as three-dimensional (3D) volumes with Imaris software (Bitplane). The Imaris Spot detection algorithm was used as described by the manufacturer for semiautomatic identification and counting of fluorescently labeled neurons. To set parameters, we manually counted enteric neurons in selected images. Additionally, images were subjected to a single Z-slice focus, then using Imaris’s Spot detection, cell count parameters were set for the size and fluorescence strength of voxels. Parameters were then subjected to multiple image tests between manually counted images and automated cell counts to ensure the accuracy of detecting only cells of interest. After validation, parameters were saved and then expedited through entire thick Z-stacks and overall cell count data were obtained for each image. The main parameters were absolute thresholding, an object size of 8-μm diameter, and a minimum “quality” score of 100. Errors in the software detection results (both erroneous positive and negative objects) were corrected by manual inspection of the data sets to adjust parameters.

### 2.6 Quantification of the immunohistochemistry staining density

The quantification of phosphorylated α-syn, CD45, or gp91^phox^ immunohistological staining in the large intestine was performed by using the colour deconvolution plugin in ImageJ software based on a protocol for unmixing hematoxylin and DAB histological stains (32). Briefly, the image was first undergoing pre-processing to ensure a neural background, then selecting H DAB vector in Color Deconvolution plugin to separate stains. The separated DAB stain next was converted into the grayscale picture. Given intestine is a large and heterogeneous structure, the selection of ROIs for α-syn pathology were mainly placed around enteric and submucosal ganglion areas, while the selection of ROIs for CD45 or gp91^phox^ were mainly placed in mucosa areas. At least 20 ROIs from each Swiss-roll section were selected for the measurement of the total pixels. The relative density of the staining was compared based on the density of the total pixels of a certain intestinal region (total pixels/area).

### 2.7 Total RNA and real-time RT-PCR analysis

Total RNA was isolated with Qiagen RNA mini kit. The quality and quantity of RNA were determined by NanoDrop (ThermoFisher Scientific, Waltham, MA, USA), with 260/280 > 1.8. Total RNA was reverse transcribed with MuLV reverse transcriptase and oligo-dT primers and real-time PCR analysis using SYBR green PCR master mix, and primers were designed with Primer3. The Ct values were used to calculate the relative expression by the 2^−△△Ct^ method and normalized with β-actin, setting control as 100%.

### 2.8 Western blotting assay

Expressions of proteins were quantified by western blot analysis as described previously (33–35). Whole tissue proteins were extracted from a segment of large intestine by using radioimmunoprecipitation assay (RIPA) lysis buffer (50 mM Tris-HCl, pH 8.0, 150 mM NaCl, 5 mM EDTA, 1% NP-40, 0.5% sodium deoxycholate, and 0.1% SDS) supplemented with protease inhibitor cocktail (Sigma-Aldrich), 1 mM phenylmethane sulfonyl fluoride (PMSF) (Sigma-Aldrich), and phosphatase inhibitor cocktails 2 and 3 (Sigma-Aldrich). Protein concentrations were determined by using the bicinchoninic acid assay (BCA, ThermoFisher). Protein samples were resolved on NuPAGE 4-12% Bis-Tris gels (Life technologies), and immunoblot analyses were performed using antibodies against phosphorylated α-syn at serine 129 (1:2000; Abcam) or α-syn (1:2000; Abcam). An antibody against β-actin or GAPDH (1:5000; Cell Signaling Technology) was included to monitor loading errors. The intensity of the western blot signals was quantitated using ImageJ software (NIH, Bethesda, MD, USA), and the densitometry analyses are presented as the ratio of protein/β-actin protein, and are compared with controls and normalized to 1.

### 2.9 Measurement of catecholamines and metabolites by HPLC

Assay samples were filtered (0.20 μm filter; ThermoFisher Scientific) and analyzed *via* HPLC with electrochemical detection as previously reported (24). Briefly, samples were separated on a C18 reverse-phase column (Hypersil ODS C18 column, ThermoFisher Scientific) with MD-TM mobile phase (ThermoFisher Scientific). Catecholamines DA and NE, precursor L-DOPA as well as catecholamine metabolites DOPAC and HVA were detected on a ThermoScientific Dionex UltiMate 3000 ECD-3000RS Electrochemical Detector (ThermoFisher Scientific) at 300 mV oxidation potential. The Chromeleon Chromatography Data System software package (ThermoFisher Scientific, version 7) quantified catecholamine (DA, NE), L-DOPA and metabolite (DOPAC, HVA) content present in each sample from the respective areas under the HPLC peaks based on defined calibration curves.

### 2.10 One-hour stool collection

Each mouse was placed in a separate clean cage and observed throughout the 60 min collection period. Fecal pellets were collected immediately after expulsion and placed in sealed (to avoid evaporation) 1.5 ml tubes. Tubes were weighed to obtain the wet weight of the stool; this was then dried overnight at 65°C and reweighed to obtain the dry weight (26, 36).

### 2.11 Accelerated Rotarod test

The accelerated Rotarod behavioral test was measured using a Rotamex device (Columbus Instruments) as previously reported (26). The parameters of the rotarod system include start speed, acceleration, and highest speed (1 rpm, accelerate 1 rpm/12 s, 50 rpm). To reach a stable performance, all mice were pre-trained on the rotarod. The training was performed on three consecutive days before the first test. On the test day, accelerating rotarod was performed. The mice underwent three consecutive trials. The rest period between each trial was 30 min. The mean latency time to fall off the rotating rod for the last two trials was used for the analysis.

### 2.12 Statistical analysis

All analysis were conducted by 2-3 investigators blind to the treatment groups and all data are reported as means ± SEM. GraphPad Prism 9 (GraphPad Software, La Jolla, CA) was used for statistical analyses. Group means were compared using ordinary one- or two-way Analysis of Variance (ANOVA) with time or treatments as the independent variable. Pairwise comparisons between group means were examined using the *post hoc* Tukey multiple comparison test among groups with significant differences. For two independent data comparisons, unpaired Student’s t test was used to determine statically significance. For all comparisons, significance was set at adjusted *p* < 0.05.

## 3 Results

### 3.1 Depletion of NE increased α-syn phosphorylation in the large intestine but not the small intestine

**Figure 1:**
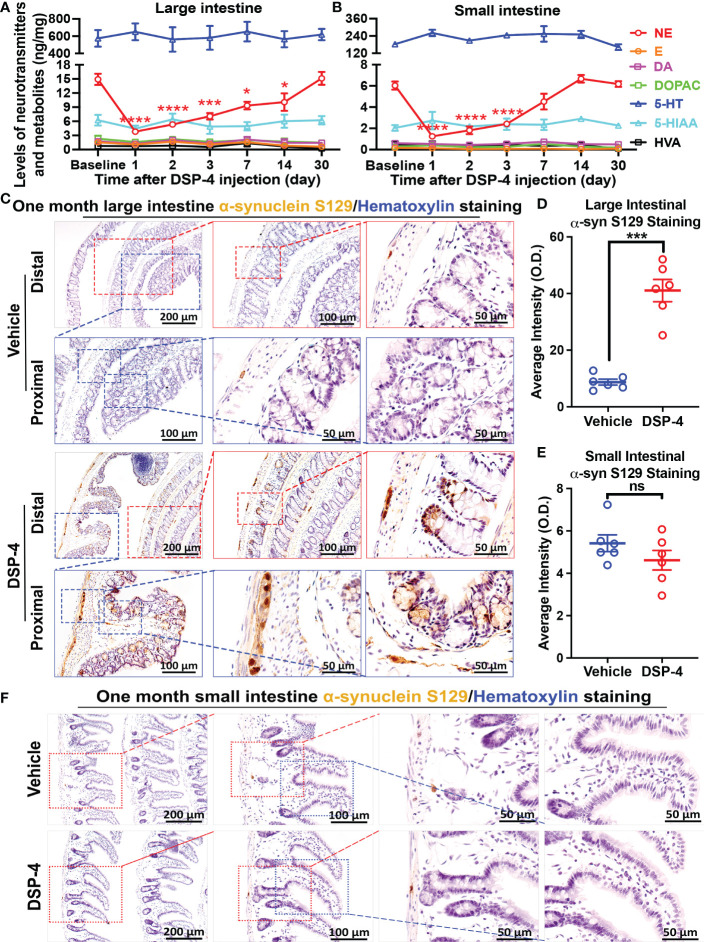
Depletion of NE increases α-syn phosphorylation in the large intestine but not the small intestine. **(A, B)** DSP-4 significantly reduced vesicular NE levels (measured by HPLC) in both large **(A)** and small **(B)** intestine. **(C–F)** Representative images **(C, F)** and the quantitative analysis **(D, E)** show increase in phosphorylated α-syn aggregation in the large intestine **(C, D)** but not in the small intestine **(E, F)** 1 month after DSP-4 injection. Statistical analysis between multiple groups using one-way ANOVA and between two groups was performed using unpaired Student’s t test. n = 5-7 mice/group. Results are expressed as mean ± SEM. *p<0.05, ***p<0.001 or ****p<0.0001 compare with baseline or age-matched vehicle controls. NE, norepinephrine; E, epinephrine; 5-HT, 5-hydroxytryptamine (serotonin); DA, dopamine. ns, not significant.

Our previous report demonstrated that depletion of brain NE by a systemic injection of DSP-4 produced a mouse PD model, which recapitulates several characteristics seen in PD patients. In this model, we observed time-dependent enhanced neuroinflammation in LC/NE innervated brain regions, followed by neuronal loss in an ascending pattern which was accompanied by the appearance of both motor and non-motor behaviors (24, 26). This study sought to investigate whether depletion of NE by DSP-4 causes early gut dysfunction by first examining α-syn pathology in both large and small intestines. We used transgenic mice over-expressing human A53T mutant α-syn (SNCA) to examine the phosphorylation of α-syn. DSP-4 caused a specific decrease in the level of NE, but not dopamine or serotonin in the gut. HPLC analysis showed a large decrease of tissue levels of NE one day after DSP-4 injection with losses of over 75% in the large intestine (p<0.001; **Figure 1A**) and 80% in small intestines (p<0.001; **Figure 1B**). Intestinal NE levels gradually recovered but remained significantly lower for up to 7 days. NE levels returned to normal by 7-14 days in the small intestine and by 30 days in the large intestine. This observation suggests that DSP-4 temporarily suppresses the production of norepinephrine, but it does not cause loss of sympathetic neurons.

Phosphorylated α-syn (p-α-syn) in Lewy bodies and Lewy neurites has been used as a biomarker to characterize the extent of neuropathology in the brains of PD patients (37, 38). We next used an anti-α-syn (pSer129) antibody to detect α-syn pathology in the gut. Interestingly, a rapid-onset of α-syn pathology was observed in enteric neurons 2 weeks after DSP-4 injection (**Supplementary Figure 1**); α-syn pathology was significantly increased at 1 month (**Figures 1C, D**) and continued to increase in a time-dependent manner (**Supplementary Figure 1**). The spectacular increase in p-α-syn-immunoreactivity was not only detected in the myenteric ganglia but also was found in colon mucosa and submucosa of DSP-4-treated animals (**Figure 1C**). By contrast, despite a similar reduction in NE level in the small intestine (**Figure 1B**), no increase in p-α-syn pathology was observed in the small intestines (**Figures 1E, F**). Possible explanations for the disparity of p-α-syn levels in large and small intestine will be discussed later.

### 3.2 Abnormal α-syn pathology was induced by post-translational modification

**Figure 2:**
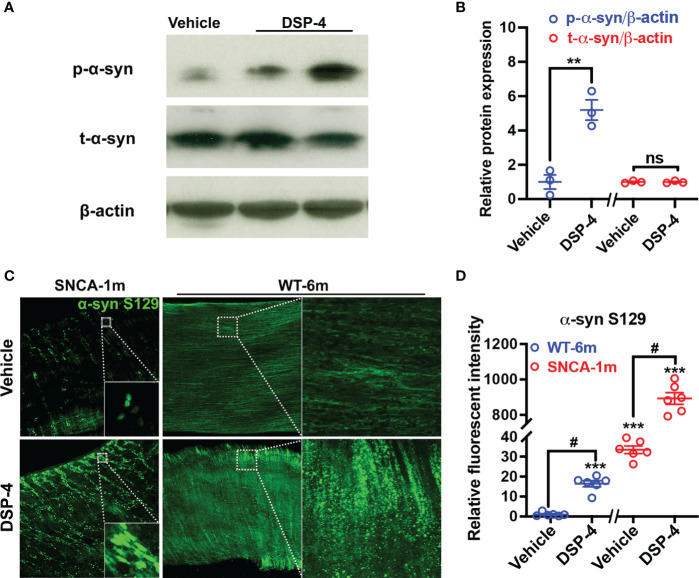
Pathological α-syn aggregation is induced by post-translational modification but not by upregulation of α-syn expression. **(A)** Representative Western blotting images show DSP-4 significantly increased α-syn phosphorylation at serine 129, while no change of total α-syn (t-α-syn) protein level was observed. **(B)** Quantitative analysis of Western blotting images for the level of p-α-syn and t-α-syn. n = 3 mice/group. **(C)** Representative immunofluorescence images show increase in α-syn phosphorylation in a segment of the large intestine in both SNCA and WT mice using CLARITY technique. **(D)** Quantitative analysis of immunofluorescence images for the intensity of p-α-syn. Statistical analysis between two groups was performed using unpaired Student’s t test. n = 6 mice/group. Results are expressed as mean ± SEM. **p<0.01, ****p<0.0001 compare with age-matched vehicle controls; ^#^p<0.05 compare with indicated groups. ns, not significant.

We further found that although the level of p-α-syn was significantly increased, the total amount of α-syn (T-α-syn) remains unchanged in the colon of NE-depleted mutant α-syn transgenic mice 1 month after DSP-4 injection (**Figures 2A, B**). Thus, post-translational phosphorylation at Ser129 was important for α-syn pathology in the colon in the two-hit model. To fully appreciate how post-translational modifications impact the α-syn pathology, we employed a protocol as reported previously for passive tissue clearing (30) to visualize the 3D distribution of p-α-syn in enteric ganglia in a segment of mouse large intestine. The robust p-α-syn immunoreactivity was clearly identified not only in myenteric but also in submucosal and mucosal plexuses in SNCA-A53T mice 1 month after DSP-4 injection (**Figures 2C, D**, **Supplementary Video 1A–D**). The p-α-syn level was also measured in WT C57BL/6 mice. Much less p-α-syn immunoreactivity was found in the WT mouse; discernible pathological aggregation of p-α-syn was not observed until 6 months after NE depletion. This is likely due to the low level of intrinsic α-syn in the WT mouse compared with the highly expressed α-syn in SNCA-A53T transgenic mice.

### 3.3 NE played a key role in enteric neurodegeneration and behavioral deficits

**Figure 3:**
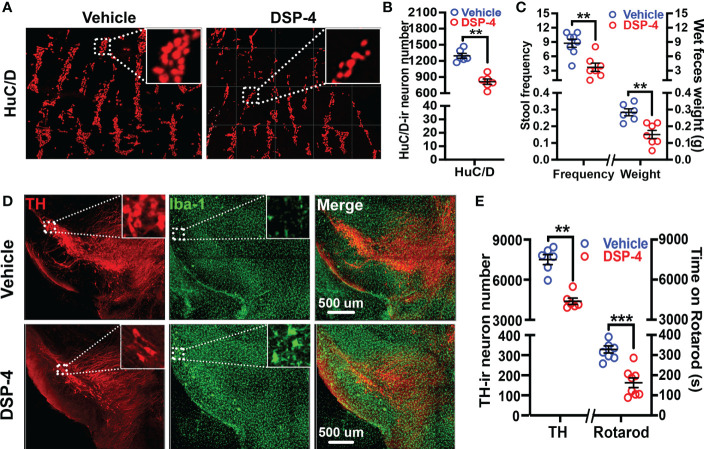
Neuronal loss and behavioral deficits occurred earlier in the colon than that in the brain after DSP-4 injection. **(A)** Representative images show neuronal loss in the colon of α-syn transgenic mice at 1 month after DSP-4 injection. **(B)** Numbers of neurons labeled with HuC/D were counted in 3 dimensional images of the cleared large intestines (**Supplementary Videos 2A, B**) by Imaris software (10 random ROIs per image were counted, ROI: X × Y × Z = 400 µm × 100 µm × 100 µm) (n = 6). **(C)** DSP-4-induced defecatory dysfunction is indicated by decreases in 1-hour stool frequency and wet feces weight at 1 month after DSP-4 injection. n = 8. **(D)** Representative images show decreased number of dopaminergic neurons (red) and increased microglial activation (hypertrophied morphology, green) in the SN of α-syn transgenic mice at 5 months after DSP-4 injection. **(E)** Quantitative analysis of dopaminergic neuronal loss in the SN (**Supplementary Videos 3A, B**) and locomotor deficits at 5 months after DSP-4 injection in α-syn transgenic mice. Statistical analysis between two groups was performed using unpaired Student’s t test. n = 6. Results are expressed as mean ± SEM. **p<0.01, ***p<0.001 compare with age-matched vehicle controls.

It is generally believed that α-syn pathology in the enteric nervous system (ENS) is related to gut dysfunction (9, 39, 40). To evaluate the loss of enteric neurons after NE depletion, cleared large intestinal tissues were stained with a pan-neuronal marker HuC/D, and the number of HuC/D-positive cells in a segment of the large intestine were semi-automatically counted using Bitplane Imaris’ spot detection algorithm in a 3-dimensional fashion (41). We found that the enteric neurons were markedly decreased in DSP-4-treated transgenic mice one month after injection (**Figures 3A, B**). Moreover, the loss of enteric neurons was associated with defecatory dysfunction, which is one of the earliest pre-motor symptoms often occurred in many PD patients. Severe constipation in SNCA-A53T mice was observed one month after DSP-4 injection, which was characterized by reduced stool frequency and decreased wet weight of feces produced within 1 hour (**Figure 3C**). By contrast, TH-staining showed no loss of nigral dopaminergic neurons in DSP-4-treated transgenic mice at one month after injection. Instead, small but significant nigral dopaminergic neurodegeneration (20% loss) was observed 3 months after DSP-4 injection (**Supplementary Figure 2**), but the mice failed to display motor deficits at this point; the impaired rotarod performance was not manifested until 5 months later when approximately 40% loss of dopaminergic neurons was reached (**Figures 3D, E**). These findings are consistent with the clinical findings that gut dysfunction occurs earlier than the motor dysfunction in PD patients.

### 3.4 Depletion of NE disrupted immune homeostasis and elicited α-syn pathology in the colon

**Figure 4:**
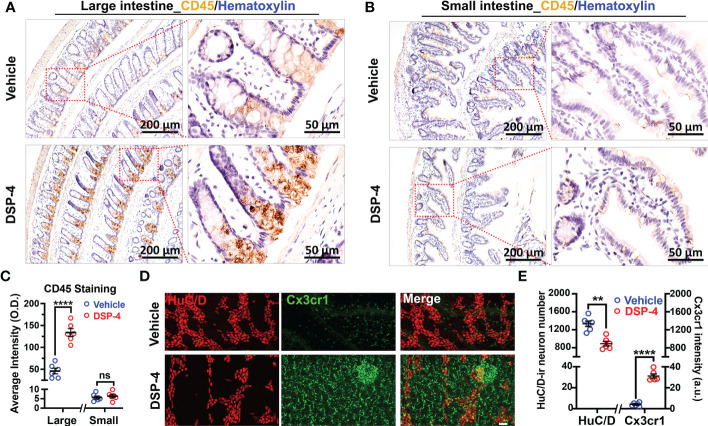
Depletion of NE disrupts intestinal immune homeostasis. **(A, B)** Increased CD45 immunoreactivity was observed in large **(A)**, but not small **(B)**, intestine of α-syn transgenic mice at 1 month after DSP-4 injection. **(C)** Quantitative analysis of CD45 intensity in large **(A)** and small **(B)** intestine. **(D, E)** GFP-Cx3cr1-immuno-positive cells from CX3CR1^+^/^GFP^ mice (green) were co-stained with pan neuronal marker HuC/D (red). The increase in intestinal immune cells was observed in different layers of the intestine, including the mucosa, submucosa and muscularis externa. The decrease in enteric neurons was observed in myenteric and submucosal plexuses (**Supplementary Videos 3A, B**). 10 random ROIs per image were counted, ROI: X × Y × Z = 400 µm × 100 µm × 100 µm, n = 6. Statistical analysis between two groups was performed using unpaired Student’s t test. Results are expressed as mean ± SEM. **p<0.01, ****p<0.0001 compare with age-matched vehicle controls. ns, not significant.

We previously reported that depletion of central NE produced a time-related increase in chronic neuroinflammation, which in turn drives progressive neurodegeneration in LC-noradrenergic neuron-innervated mouse brain regions (24, 26). Indeed, NE depletion by DSP-4 induced a significant increase in microglial intensity (Iba-1 staining) and activation (hypertrophied morphology) in the SN (**Figure 3D**). To further understand the role of NE in maintaining immune homeostasis in the gut, we stained colon tissues with leucocyte common antigen (CD45), a general leukocyte marker in DSP-4-injected mice. A higher level of CD45-immunoreactivity was found in the colon than that in the small intestine in control mice. However, the greatly enhanced immunoreactivity of CD45-positive cells was only observed in the mucosa of the large (**Figure 4A**), but not the small (**Figure 4B**), intestine one month after DSP-4 injection. Consistently, the abundant abnormal p-α-syn pathology was only found in the large intestine (**Figures 1C–F**); the increased mucosal immune activity was also only presented in the large intestine but not in the small intestine 1 month after DSP-4 injection (**Figures 4A–C**).

The results that the depletion of NE induced a significant increase in immune cells and decrease in enteric neurons were further validated in cleared intestinal tissues in DSP-4-treated CX3CR1^+^/^GFP^ mice at 6 months (**Figures 4D, E**). This transgenic mouse strain enabled us to visualize all GFP labeled monocytes, subsets of NK cells, dendritic cells, and other immune cells in the gut (42). The 3D video clearly showed that the increased immune cells were mainly located in mucosal region of the large intestine (**Supplementary Videos 4A, B**). Altogether, these findings suggest that the large intestine is preferentially affected in the NE-depleted mouse model of PD.

### 3.5 NE depletion increased the expression of chronic inflammatory genes in the gut

**Figure 5:**
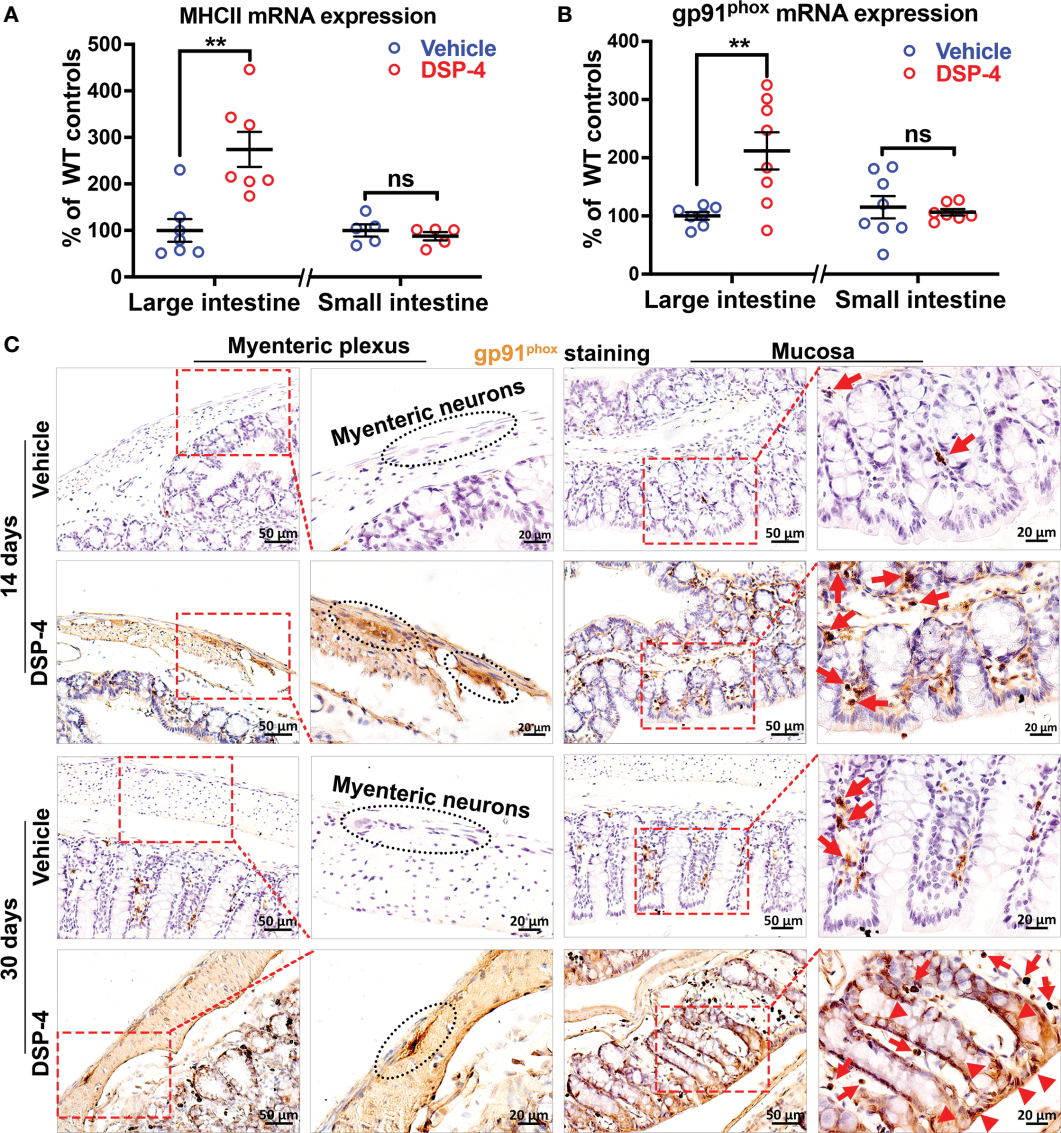
NE depletion increased the expression of chronic inflammatory genes in gut. **(A, B)** Increased expression of chronic inflammatory genes MHCII **(A)** and gp91^phox^ **(B)** in large intestine of α-syn transgenic mice 1 month after DSP-4 injection. n = 7 mice/group. **(C)** The high-power images show gp91^phox^ was found initially only in immune cells (arrows) during the acute intestinal inflammation stage (14 days, upper panel); intense gp91^phox^-immunostaing was also found in enteric neurons and epithelial cells (arrow heads) during the stage of chronic inflammation (30 days, lower panel). Statistical analysis between two groups was performed using unpaired Student’s t test. Results are expressed as mean ± SEM. **p<0.01 compare with age-matched vehicle controls. ns, not significant.

To further investigate DSP-4-induced chronic intestinal inflammation, we measured expression of two important immune genes, MHCII (**Figure 5A**) and gp91^phox^ (**Figure 5B**) in the large intestine after 1 month of DSP-4 injection. These two genes are closely related to the formation and maintenance of chronic inflammation in the mouse brain after either LPS or DSP-4 injection (27). MHC II molecules are normally found on antigen-presenting cells, which are important in initiating adaptive immune responses when inflammation becomes chronic. Whilst microglial gp91^phox^ (NOX2), the catalytic membrane subunit of NADPH oxidase that is a key superoxide-producing enzyme. Once activated, it produces extracellular superoxide free radical and increases intracellular reactive oxygen species (ROS) that are critical in initiating and maintaining chronic inflammatory responses. Again, the increased immunoreactivities of both gp91^phox^ and MHCII were found in the large, but not the small intestine.

Moreover, it’s interesting to note that the increase in gp91^phox^ expression was found initially in immune cells and enteric neurons in the early inflammatory stage (**Figure 5C**, upper panel, arrows). However, the intense gp91^phox^ expression was also found in epithelial cells when inflammation becomes chronic (**Figure 5C**, bottom, arrow heads).

### 3.6 Depletion of NE enabled α-syn pathology to start in the intestinal lumen *via* upregulation of NOX2 in both enteroendocrine and goblet cells

**Figure 6:**
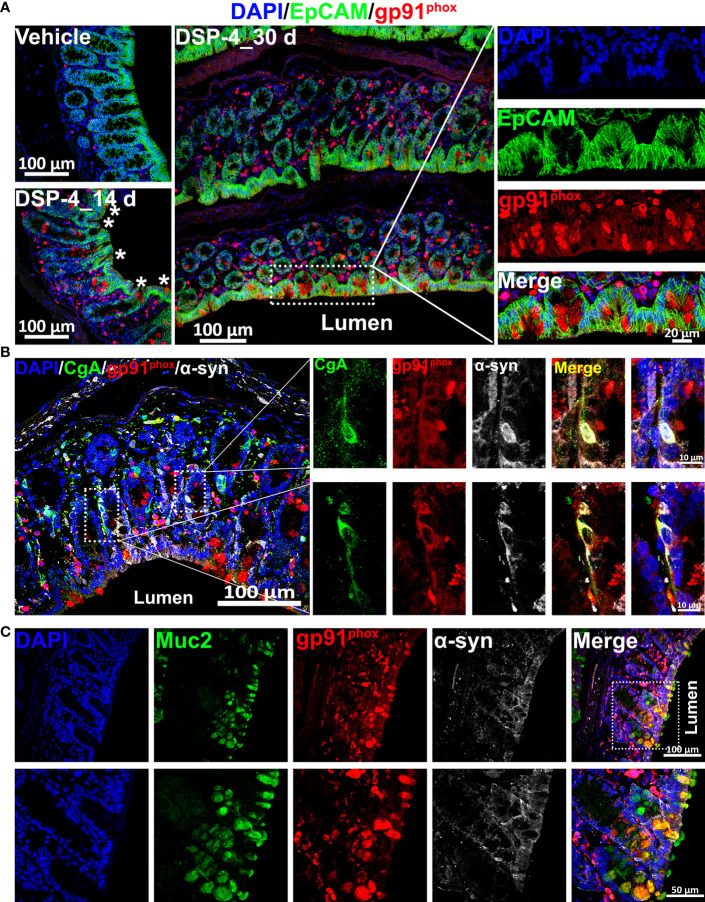
NE depletion results in an increase in the level of NOX 2 in both enteroendocrine and goblet cells. **(A)** The large intestine of α-syn transgenic mice is double-labeled for EpCAM (epithelial cell marker, green) and gp91^phox^ (red) with DAPI nuclear counterstain (blue). The images show increase of gp91^phox^-positive cells in the large intestine 14 and 30 days after DSP-4 injection. **(B)** α-syn (white) and gp91^phox^ (red) are co-expressed in enteroendocrine cells (EECs) of mouse colon 30 days after DSP-4 injection. EECs are identified by immunostaining for chromogranin A (CgA; green). Two representative EECs (boxed) are shown at higher magnification at right. Both α-syn and gp91^phox^ are presented inside the EECs. Scale bar: 100 µm (left); 10 µm (right). **(C)** DSP-4-treated large intestines of α-syn transgenic mice (30 days) were double-stained with gp91^phox^ (red) and Muc2 (green). The results demonstrate that the increased gp91^phox^-positive cells on the innermost layer of intestinal barrier are goblet cells. Scale bar: 100 µm (top); 50 µm (bottom).

Given that chronic inflammation results in an excess of ROS that are able to post-transnationally modify proteins, such as α-syn (43, 44), the distribution of gp91^phox^-positive cells, especially those were scattered among the innermost epithelial lining of the large intestine (**Figures 5C**, **6A**) may play a key role in regulating immune homeostasis in the gut. The gate-keeping function of those gp91^phox^-positive epithelial cells drew our attention to enteroendocrine cells (EECs) and goblet cells. Triple immunofluorescence analysis showing that those gp91^phox+^ cells are goblet cells (**Figure 6C**) rather than EECs (**Figure 6B**). Interestingly, all the gp91^phox+^/Muc2^+^ goblet cells were located on mucosal epithelial barrier and showed immune cell-like property (**Figure 5C**). Although the EECs marker chromogranin A (CgA) is co-localized with gp91^phox^, none of them was detected on the mucosal epithelial barrier (**Figure 5B**). EECs also presented a neuron-like property, as they colocalized with α-syn staining, which suggests that the p-α-syn positive immunoreactivity on mucosal region (**Figure 1C**) is partially come from EECs, besides enteric nerves. Since goblet cells are well appreciated in maintaining epithelial barrier through the secretion of mucus (45, 46), the increase in gp91^phox^ expression and production of superoxide and its downstream ROS will cause oxidative damage, compromise the barrier and allow for the encroachment of the microbiota and foreign antigens from the luminal compartment. Eventually, disruption of mucosal immune homeostasis occurs and leads to α-syn pathology from EECs to enteric nerve system.

### 3.7 Depletion of NE in gut increased oxidative damage to intestinal barrier and enteric neurons

**Figure 7:**
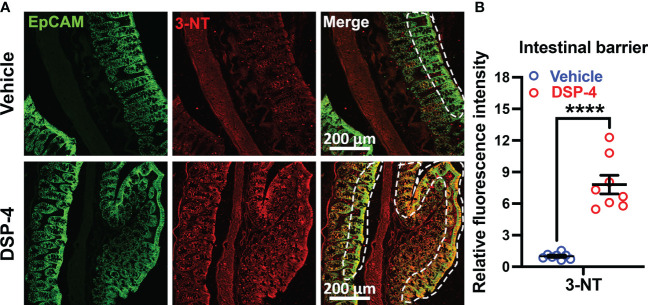
Male SNCA-A53T mice received a single injection of DSP-4 (50 mg/kg, i.p), oxidative proteins in large intestines were stained with anti-3-NT antibody. **(A)** Representative images show double labeled epithelial cell adhesion molecule (EpCAM) and 3-NT in large intestines 1 month after DSP-4 injection. Images were captured at 40X magnification. **(B)** The relative fluorescence intensity of 3-NT-immunoreactivity in the epithelial barrier was quantified. Statistical analysis between two groups was performed using unpaired Student’s t test. n = 8. Results are expressed as mean ± SEM. ****p<0.0001.

We next examined whether the continuous ROS generation by both immune cells and epithelial cells at the site of inflammation could cause excessive oxidative stress and produce tissue injury. We found that the oxidative stress of epithelial cells (ECs) is significantly increased in the large intestine in mice one month after DSP-4-injection (**Figure 7**), as evidenced by Epcam and 3-NT (oxidative stress marker) double immunolabeling. The innermost layer of mucosa is a single layer of epithelial cells (ECs). They act as a barrier to prevent the passage of harmful intraluminal substances including foreign antigens, microorganisms and their toxins. The increased immune response (**Figure 4**) and gp91^phox^ expression on enteroendocrine and goblet cells (**Figures 5C**, **6**), facilitates recruitment and infiltration of immune cells at the mucosal area (**Figures 4**–**6**), resulting in chronic mucosal inflammation and an increase in oxidative damage to the epithelium (**Figure 7**) and subsequent intestinal barrier dysfunction. The mucosal barrier outlined by white dotted lines (**Figure 7A**) showed inflamed epithelium, not only highlighting localized 3-NT staining in intra-epithelial lymphocytes, but also showed a disturbed cellular morphology with goblet-cell hyperplasia and dysregulated mucus attachment (**Supplementary Figure 3**). Depletion of NE also showed a greatly neuronal oxidative stress on enteric neurons (**Supplementary Figure 4**).

### 3.8 Post-treatment with salmeterol or DPI prevented DSP-4-induced gut pathologies in SNCA-A53T mice

**Figure 8:**
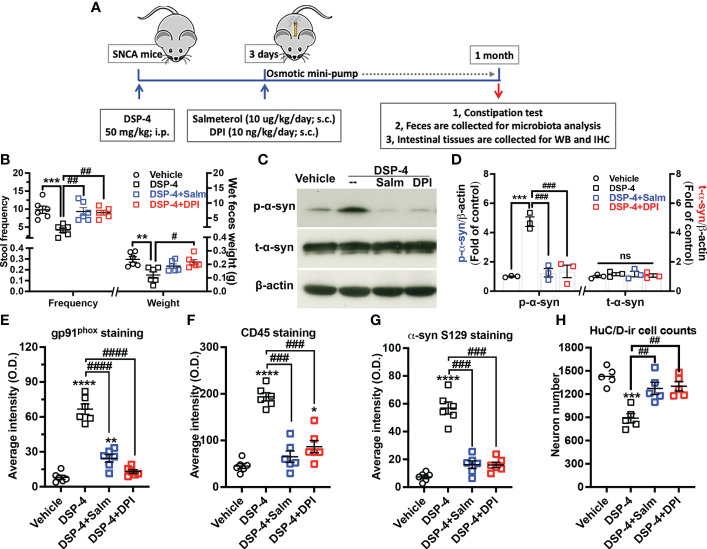
Post-treatment with salmeterol or DPI prevents DSP-4-induced gut pathologies in SNCA-A53T mice. **(A)** Dosing regimen. **(B)** Both salmeterol (10 µg/kg/day) and DPI (10 ng/kg/day) markedly improved DSP-4 induced defecatory dysfunction. n = 6. **(C, D)** Representative Western blotting image **(C)** and qualitative analysis (**D**, n = 3) of p-α-syn S129 and t-α-syn protein. **(E–G)** Quantitative analysis of gp91^phox^, CD45 and p-α-syn S129 immunostaining intensity in the experiments using mini-pumps (**Supplementary Figure 5**). **(H)** Number of neurons labeled with anti-HuC/D antibody in 3 dimensional images was conducted in cleared large intestines by using Imaris software (ROI: X × Y × Z = 400µm × 100µm × 100µm) (n = 6). Statistical analysis between multiple groups using one-way ANOVA followed by Tukey’s multiple comparisons test performed. Results are expressed as mean ± SEM. **p<0.01, ***p<0.001 or ****p<0.0001 compare with age-matched vehicle controls; ^#^p<0.05, ^##^p<0.01, ^###^p<0.001 or ^####^p<0.0001 compare with indicated groups. Salm, salmeterol. ns, not significant.

To obtain additional evidence supporting the critical role of inflammation in driving gut α-syn pathology and to develop potential therapy, two anti-inflammatory pharmacological reagents were used in DSP-4 injected mice. Salmeterol, a long-acting β2-AR agonist, was used to restore norepinephrine transmission in NE depleted gut. DPI is a widely used NOX2 inhibitor. Two days after DSP-4 injection, SNCA-A53T mice received either salmeterol (10 µg/kg/day) or DPI 10 (ng/kg/day) for 28 days through osmotic mini-pump inserted subcutaneously (**Figure 8A**). Four weeks after DPI or salmeterol post-treatment, both interventions greatly improved DSP-4-induced defecatory dysfunction in stool frequency and wet feces weight (**Figure 8B**) and significantly reduced the level of p-α-syn protein in the large intestine (**Figures 8C, D**). Furthermore, both reagents effectively ameliorated DSP-4-induced gut inflammation, as shown by decreased CD45- and gp91^phox^-immunoreactivity (**Figures 8E, F**), reduced α-syn pathology (**Figure 8G**), and attenuated enteric neuronal loss (**Figure 8H**) in the large intestine.

## 4 Discussion

Using a “two-hit” model (neurotoxin combined with transgenic expression of mutant human α-syn), we demonstrate that depletion of intestinal NE content by DSP-4 produced gut pathology about 3-5 months ahead of the onset of brain neurodegeneration in A53T-SNCA mice. Pronounced colon inflammation was first observed, followed by a large increase in the immunoreactivity of p-α-syn at serine-129 and loss of enteric neurons in the colon but not in the small intestine. As a result of these colon dysfunctions, DSP-4 injected mice showed constipation. Mechanistic studies reveal that DSP-4-elicited upregulation of NOX2 was initially only occurred in immune cells during the acute intestinal inflammation stage. The intense NOX2-immunoreactivity was also observed in enteric neurons and mucosal epithelial cells during the stage of chronic inflammation. The increase in NOX2 expression correlated well with the extent of p-α-syn aggregation and subsequent enteric neuronal loss, suggesting that NOX2-derived ROS play a key role in α-synucleinopathy. Taken together, our “two-hit” model of PD shows a progressive pattern of pathological changes from the gut to the brain and suggests a potential role of dysfunction of the noradrenergic system in the pathogenesis of PD.

### 4.1 Dysfunction of the noradrenergic system produces numerous cardinal premotor symptoms of PD

Although dysfunction of the NE system has been closely linked with some of the premotor symptoms of PD (47, 48), NE-related animal PD models have yet to be fully studied. Our previous work showed that DSP-4-injected mice not only exhibited sequential caudo-rostral neurodegeneration in the brain but also displayed time-dependent nonmotor and motor dysfunctions (24, 26). This study added additional important data to show that depletion of peripheral NE levels produced prominent GI dysfunctions, such as colon inflammation, α-synucleinopathy, enteric neuron loss, and constipation. The colon pathologies may link to the changes of microbiota in DSP-4 injected mice (27). Taken together, results from these studies provide further evidence supporting the role of NE in the pathogenesis of PD.

### 4.2 Why α-syn pathology was found only in the large intestine but not in the small intestine?

One of the interesting findings from this study was the regional differences in the GI tract affected by DSP-4 injection. While the colon displayed robust pathologies after DSP-4 treatment, the entire small intestine showed little changes. There are several possible explanations for these discrete regional differences.

1) The content of NE in the colon is about 2.5-fold higher than that in the small intestine (**Figures 1A, B**), suggesting higher innervation of adrenergic neurons in the colon than in the small intestine. Although the degree of decrease of the NE content (in percentage) exerted by DSP-4 is comparable in both regions, a greater loss of absolute NE values was found in the colon. Moreover, it takes a longer time for NE to return to the control level in the colon than in the small intestine. Given the potent anti-inflammatory property of NE (25), the less amount of NE released from the adrenergic neurons renders macrophages in the colon more prone to activation and inflammation.
2) Our data showed that numbers of mucosal CD45-positive cells are higher in the colon than in the small intestine in untreated mice (**Figure 4**). Depletion of NE by DSP-4 enhances the recruitment of a variety of immune cells and greatly increases the number of leucocytes in the colon.
3) The colon harbors a large and complex community of microbiota (more than 90% of the microbiota in the GI tract), which is a major source of many essential chemical factors that drive normal or abnormal metabolism (49). These organisms, together with the antigenic load provided by the diet and the constant threat of potential pathogens, makes the intestinal immune system encounter more antigen than any other part of the body. Chronic colonic mucosal inflammation can alter the composition of microbiota favoring the growth of pathogenic species, which may release pro-inflammatory substances to aggravate colonic mucosal inflammation (50). The altered gut microbiota has been demonstrated playing an important role in regulating GI dysfunction and motor symptoms in PD (20–22).

### 4.3 NE depletion in the gut is necessary to produce DSP-4-elicited colon pathology

Similar to NE depletion time course pattern in the gut, we’ve previously reported that the decrease of tissue levels of NE was also observed one day following DSP-4 injection in the brain of WT mice. But brain NE levels remained significantly reduced during the first few months, then returned to normal values by 10 months post-injection (24). It’s worth noting that while total NE tissue levels are reduced by DSP-4, extracellular NE levels and NE turnover (ratio of MHPG: NE measured by HPLC) can be normal or increased in the brain, suggesting adaptations that maintain NE transmission and homeostasis (51, 52). Unfortunately, our HPLC system failed to measure MHPG or 3MT precisely. Therefore, we are not able to comment on this point.

It is generally believed that the “gut-brain axis” is a two-way interaction. Given DSP-4 decreases NE levels both in the brain and in the gut, a question arises as to whether the brain plays a role in DSP-4-elicited colon pathologies. To answer this question, we performed a separate experiment by using a conditional dopamine-beta hydroxylase-(DBH) knockout mouse strain (in collaboration with Dr. Patricia Jensen, NIEHS/NIH) in which depletion of NE from adrenergic neurons occurs only in the brain, but not in the peripheral organs. This strain of mice displays profound loss of brain nigral dopaminergic neurons and hippocampal neurons at the ages of 8-10 months, but no pathological change was observed in the gut (Song et al, unpublished data). Thus, reduction in NE levels in the gut is necessary for generating colon pathology.

### 4.4 How does depletion of gut NE lead to colon pathologies?

**Figure 9:**
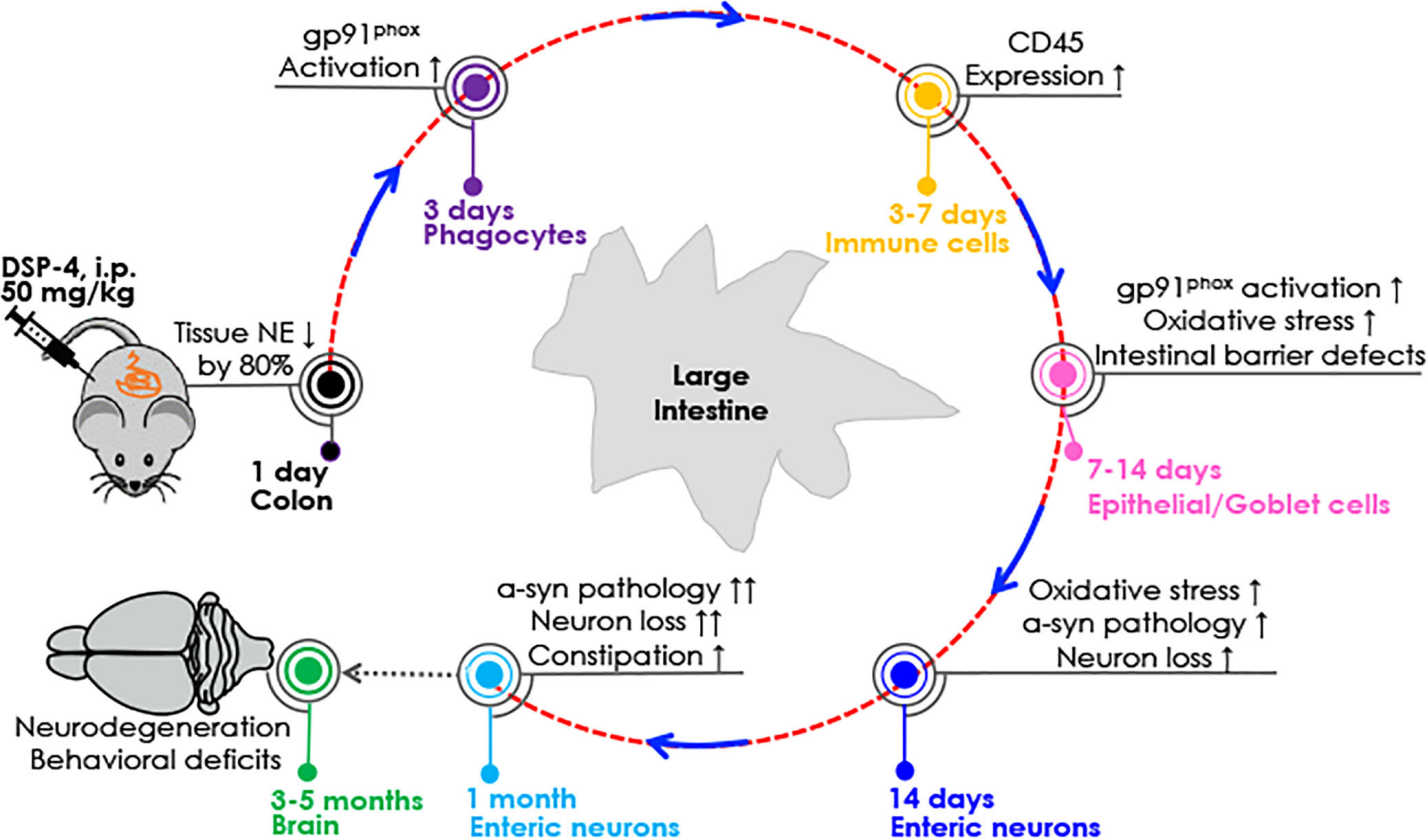
Sequential pathological events occur in the colon after DSP-4 injection. Reduction in NE levels may render the colon immune cells (e.g., macrophages, neutrophils, and lymphocytes) more sensitive to immune stimuli (such as microbiome) and easier to become activated due to the loss of tonic inhibition, producing more proinflammatory factors to cause colon inflammation. Our results suggest that progressive intestinal inflammation leads to enhanced activation and expression of NOX2 and oxidative stress, which in turn augments α-syn aggregation/propagation and finally drives neurodegeneration and behavioral deficits in the gut. For detailed information, see the discussion.

Many neurotransmitters produced locally in the gut, such as NE, dopamine, 5-HT (serotonin), GABA, and acetylcholine play regulatory roles in gut motility and immune homeostasis (53–57). Among the biogenic amines measured by HPLC analysis, DSP-4 injection selectively reduced the NE level without affecting the levels of DA or 5-HT in the colon (**Figures 1A, B**). These data suggest that loss of NE plays a crucial role in initiating inflammation in the colon. Our data suggest that colon inflammation plays a pivotal role in the subsequent changes in colon functions, also lend credence to the idea that intestinal inflammation is a silent driver of PD pathogenesis (8, 19, 58). The discussion below will focus on several key players involved in the genesis of colon pathologies (**Figure 9**).

#### 4.4.1 NE and chronic intestinal inflammation

This study showed that following the injection of DSP-4, one of the earliest gut pathologies was colonic mucosal inflammation. Signs of inflammation occurred within a few days after NE depletion, then followed by enhanced CD45-immunoreactivity. DSP-4-elicited colon inflammation was temporarily progressive and lasted up to 1 year. The timeline study indicated that colon inflammation was followed by the increase in enteric α-syn pathology and neuronal loss. These results strongly suggest that inflammation plays a key role in driving colon pathology.

#### 4.4.2 Molecular mechanism underlying DSP-4 elicited colon pathologies

**Figure 9** depicts possible time-related events that occur in the colon after DSP-4 injection. The first question to address is how DSP-4 elicited reduction in gut NE levels causes colon pathologies. It is known that besides functioning as a neurotransmitter, NE has also been well known in the periphery for its tonic anti-inflammatory capacities (59–62). We hypothesized that DSP-4-elicited reduction in NE levels may render the colon immune cells, such as macrophages and neutrophils more sensitive to immune stimuli (such microbiome) and easier to become activated due to the loss of tonic inhibition.

#### 4.4.3 NOX is critical in mediating colon inflammation

Many reports indicate that the anti-inflammatory effect of NE is mediated through β-adrenergic receptors (28, 63–65). However, our laboratory has previously reported a new β-adrenergic receptor-independent target for the actions of NE and salmeterol, a long-lasting β2-AR agonist. *In vitro* studies showed that lower concentrations of NE (10 nM) or salmeterol (1 nM), which are not sufficient to activate β-adrenergic receptors, exert potent anti-inflammatory and neuroprotective effects in mouse primary neuron-glial cultures through a NOX2-dependent signaling pathway (25). In this study, we found a critical role of superoxide-producing enzyme NOX2 in colon inflammation by demonstrating a significant increase in the expression of gp91^phox^ (NOX2) in the colon (**Figure 5C**) and protective effects of NOX2 inhibitor DPI (**Figure 8**).

It has been reported that NOX1 and NOX2 are the two major subtypes of NOX found in the colon, where NOX1 is mainly expressed on colonic epithelial cells and NOX2 is highly expressed on macrophages and neutrophils (66). Superoxide and related ROS have been shown to play critical roles in chronic inflammation-related GI diseases such as irritable bowel diseases, constipation, and rectal cancer (67, 68). Given the crucial role of macrophages and neutrophils in mediating antimicrobial host defense and in coordinating the inflammatory response in gut, the attention of this study has focused on NOX2. Moreover, it is of particular interest to note that goblet cells, specialized epithelial cells that serve as single-cell glands and line multiple mucosal surfaces, also displayed high level of NOX2-immunoreavtivity after DSP-4 injection. Goblet cells are well appreciated in maintaining epithelial barrier through the synthesis and secretion of mucus (45, 46). The abnormal increase in gp91^phox^ expression could cause oxidative damage of cells and compromise the barrier, thus allowing for the encroachment of the pathogens, microbiota and foreign antigens from the luminal compartment, disrupting mucosal immune homeostasis and eventually leading to mucosal inflammation and α-syn pathology.

The mucus layer coating the GI tract is the front line of innate host defense. The mucus secretion is increased in the face of threats, and increased mucus secretion has also been demonstrated to be the cause of severe ER stress and apoptosis of goblet cells that preceded absorptive epithelial cell damage (69). Since β2-AR was distinctively expressed in numerous intestinal-type crypts and goblet cells (70, 71), it’s reasoned that goblet cells are one of the first cells to respond to NE-depleting in the gut, and play a critical role in initiating α-syn pathology. Thus, in GI inflammation, a dysfunctional epithelial barrier could be ascribed to the high mucus biosynthesis and goblet cell apoptosis, which aligns with our finding that depleting of NE by DSP-4 accelerates the biosynthesis and secretion of MUC2 in the large intestine (**Supplementary Figure 3**).

#### 4.4.4 Oxidative stress mediates chronic inflammation-elicited gut pathologies

We have observed a large increase in oxidative stress marker 3-NT immunoreactivity in different cell types of the colon tissue in DSP-4 injected mice (**Figure 7**). This is related to the overproduction of superoxide and its metabolites ROS from prolonged activation of NOX in immune cells and mucosal epithelial cells (ECs). Excessive oxidative stress could produce a series of tissue damage and pathologies. Oxidative damage of ECs is significantly increased in the large intestine which functions as a barrier to prevent the passage of harmful intraluminal substances including foreign antigens, microorganisms, and their toxins. These results suggest that the epithelial tight junctions have been compromised by chronic mucosal inflammation (**Figure 6**). Chronic mucosal inflammation also affects the gut microbiota balance. We have recently reported that DSP-4 injection alters gut microbiota composition: the increased abundance of Verrucomicrobiaceae and decreased abundance of Prevotellaceae (27). Furthermore, enhanced oxidative stress could also underlie the formation of α-synucleinopathy through the increase of phosphorylated α-syn (43, 44).

In addition to enteric neurons, a great amount of phosphorylated α-syn was found in mucosal and sub-mucosal areas, where we identified some are on enteric nerves (**Figure 1C**, lower panel), but most of them are on epithelial cells (**Figure 1C**), specifically on enteroendocrine cells (EECs, **Figure 6B**). Those findings align with a recent study that reported EECs possess many neuron-like properties and express α-syn (72). Moreover, the α-syn–containing EECs also expressed high levels of gp91^phox^ (**Figure 6B**). Given the α-syn–containing EECs directly connect to enteric nerves, it lends support to the assumption that oxidative damage of intestinal mucosal barrier (goblet cells and EECs) elicited by proinflammatory insults after NE-depleting facilitate α-syn misfolding in the EECs and its propagation from the gut epithelium to the brain in a prion-like fashion through the enteric nerve system, suggesting the possibility that PD starts in the intestinal lumen.

### 4.5 Potential strategies for colon inflammation therapy

Post-treatment with a low dose of salmeterol almost completely inhibits DSP-4-elicited colon inflammation and restores GI functions (**Figure 8**). This finding adds to a long list of beta-adrenergic therapies in a variety of inflammation-related diseases. However, we speculate that the beneficial effect of salmeterol may not be mediated totally through the β2-AR. The dosage of salmeterol used in this study (10 μg/kg/day for 28 days; sc) is about one-hundredth of the regular dose commonly used in the literature (73, 74). Our previous report indicated that this low dose of salmeterol protected nigral dopamine neurons in both MPTP and LPS mouse PD modes through inhibition of NOX2 (28). Protective effects of NOX2 inhibition were further confirmed by 28-day infusion of ultralow dose DPI (10 ng/kg/day; sc), which was reported to selectively inhibit NOX2 activity and devoid of any effect on other flavin-containing oxidases (24). DPI effectively attenuates DSP-4-elicited colon pathology (**Figure 8**). Thus, NE depletion is linked to NOX2-mediated mucosal inflammation and risk of α-syn propagation in a gut-brain fashion and constitutes a potential target for therapies.

Recent data have also discovered that β2AR is an important regulator of the α-syn gene (SNCA) expression (75). Over 11 years of follow-up in 4 million people, the clinical study regarding associations of β2AR with neuronal α-syn expression and risk of PD has yielded great results in Norway. Norwegians who were prescribed salbutamol, one of the most commonly used β2AR agonists typically prescribed for asthma, show decreased risk of developing PD; the individuals who used β2AR propranolol, commonly used for hypertension, at least 365 defined daily doses show markedly increased risk of PD. Together, these findings provide strong evidence further supporting the critical role of the noradrenergic system in the pathogenesis of PD.

## 5 Conclusions

This study provides the first evidence showing that reduction of intestine NE level causes intense colon inflammation, α-syn pathology, neuronal loss, and constipation. These data not only demonstrate that NE plays a key role in regulating colon immune homeostasis but also lend credence to the concept of the gut-brain axis in PD. Moreover, our results provide insights into potential strategies for treating colon inflammation-related pathologies by targeting NOX2.

## Data availability statement

The raw data supporting the conclusions of this article will be made available by the authors, without undue reservation.

## Ethics statement

The animal study was reviewed and approved by National Institutes of Environmental Health Sciences Animal Care and Use Committee.

## Author contributions

SS and J-SH conceived and designed the experiments. SS performed the experiments. J-SH, H-MG and Y-YS supervised work, assessed data quality and contributed to the interpretation of data and statistical analyses. DT performed the western blotting experiments and analyzed the data. JL and SS performed RT-PCR, behavioral tests and analyzed the data. CM conducted immunohistochemistry analysis with BW and QW. SS drafted the manuscript with JL, Y-YS, H-MG and J-SH. SS, H-MG, Y-YS and J-SH proofed the manuscript. All authors contributed to the article and read and approved the submitted version of the manuscript. All authors contributed to the article and approved the submitted version.
