# Supplementary material for: Dysfunction of the noradrenergic system drives inflammation, α-synucleinopathy, and neuronal loss in mouse colon
Source: Front Immunol. 2023 Feb 10;14:1083513. doi: 10.3389/fimmu.2023.1083513 (PMC9950510; doi:10.3389/fimmu.2023.1083513)
Supplement: Supplementary file 1 [file Presentation_1.pptx]

## Slide 1
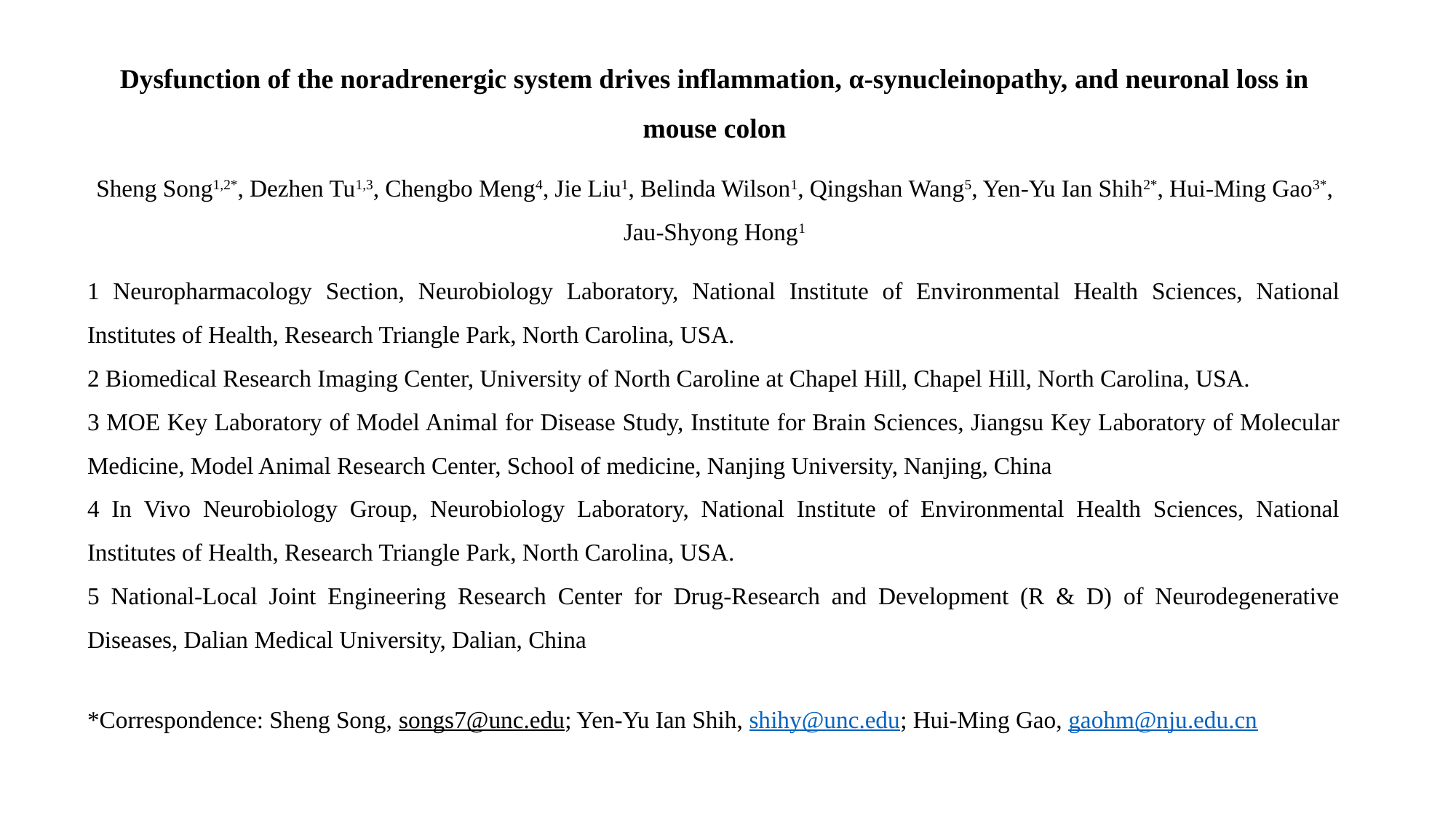

Dysfunction of the noradrenergic system drives inflammation, α-synucleinopathy, and neuronal loss in mouse colon
Sheng Song1,2*, Dezhen Tu1,3, Chengbo Meng4, Jie Liu1, Belinda Wilson1, Qingshan Wang5, Yen-Yu Ian Shih2*, Hui-Ming Gao3*, Jau-Shyong Hong1
1 Neuropharmacology Section, Neurobiology Laboratory, National Institute of Environmental Health Sciences, National Institutes of Health, Research Triangle Park, North Carolina, USA.
2 Biomedical Research Imaging Center, University of North Caroline at Chapel Hill, Chapel Hill, North Carolina, USA.
3 MOE Key Laboratory of Model Animal for Disease Study, Institute for Brain Sciences, Jiangsu Key Laboratory of Molecular Medicine, Model Animal Research Center, School of medicine, Nanjing University, Nanjing, China
4 In Vivo Neurobiology Group, Neurobiology Laboratory, National Institute of Environmental Health Sciences, National Institutes of Health, Research Triangle Park, North Carolina, USA.
5 National-Local Joint Engineering Research Center for Drug-Research and Development (R & D) of Neurodegenerative Diseases, Dalian Medical University, Dalian, China
*Correspondence: Sheng Song, songs7@unc.edu; Yen-Yu Ian Shih, shihy@unc.edu; Hui-Ming Gao, gaohm@nju.edu.cn

## Slide 2
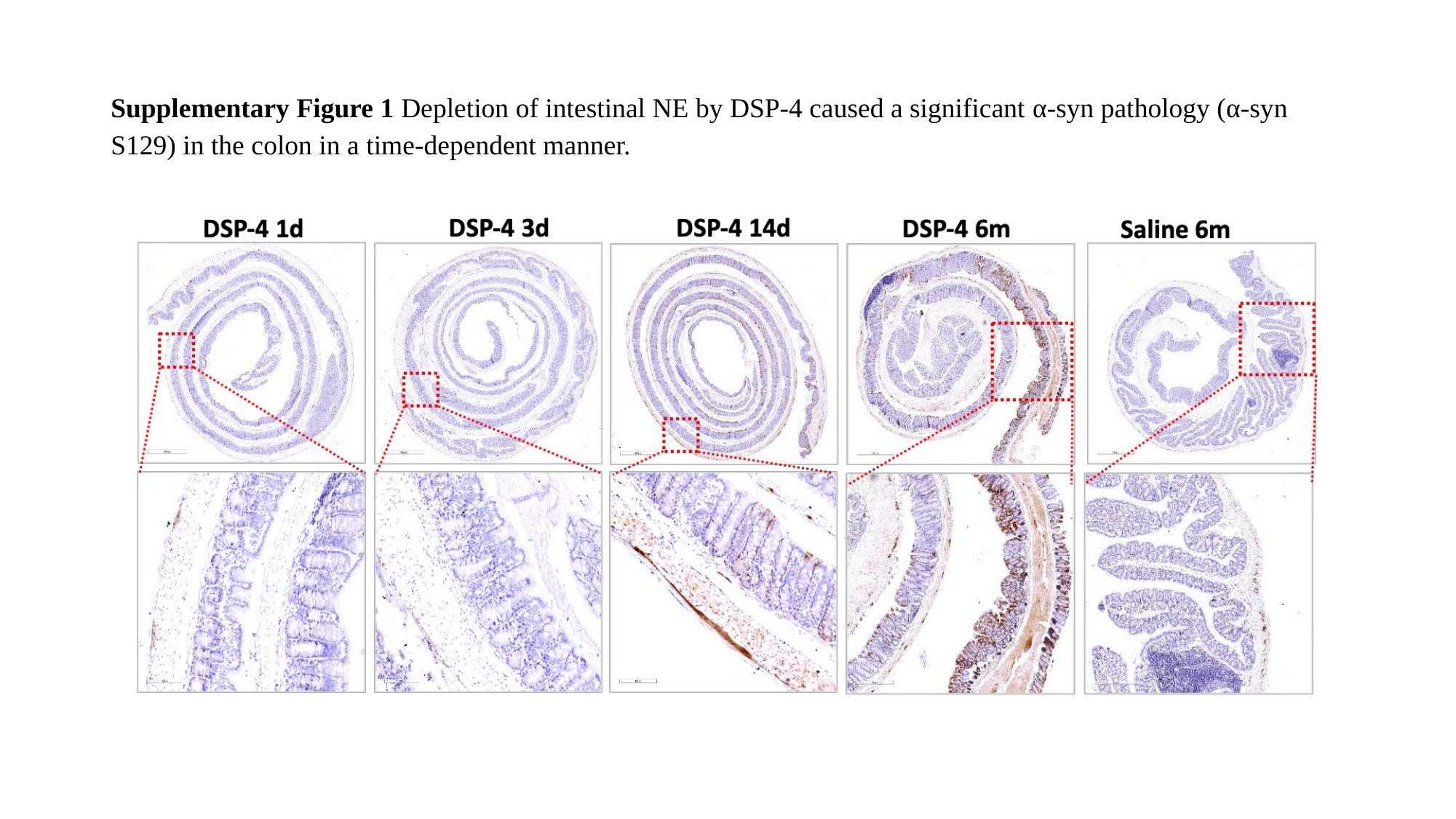

# Supplementary Figure 1 Depletion of intestinal NE by DSP-4 caused a significant α-syn pathology (α-syn S129) in the colon in a time-dependent manner.

## Slide 3
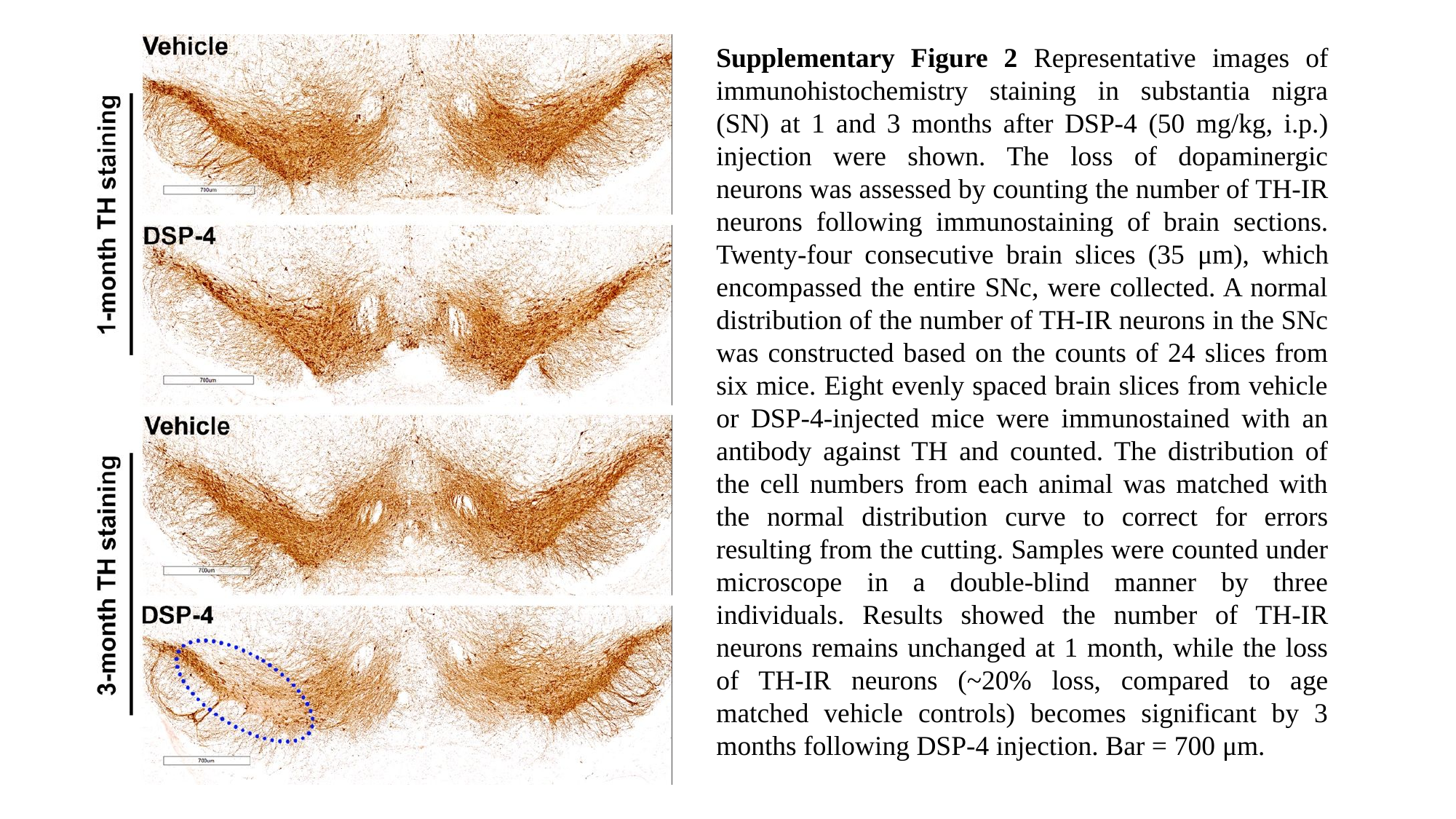

Supplementary Figure 2 Representative images of immunohistochemistry staining in substantia nigra (SN) at 1 and 3 months after DSP-4 (50 mg/kg, i.p.) injection were shown. The loss of dopaminergic neurons was assessed by counting the number of TH-IR neurons following immunostaining of brain sections. Twenty-four consecutive brain slices (35 μm), which encompassed the entire SNc, were collected. A normal distribution of the number of TH-IR neurons in the SNc was constructed based on the counts of 24 slices from six mice. Eight evenly spaced brain slices from vehicle or DSP-4-injected mice were immunostained with an antibody against TH and counted. The distribution of the cell numbers from each animal was matched with the normal distribution curve to correct for errors resulting from the cutting. Samples were counted under microscope in a double-blind manner by three individuals. Results showed the number of TH-IR neurons remains unchanged at 1 month, while the loss of TH-IR neurons (~20% loss, compared to age matched vehicle controls) becomes significant by 3 months following DSP-4 injection. Bar = 700 μm.

## Slide 4
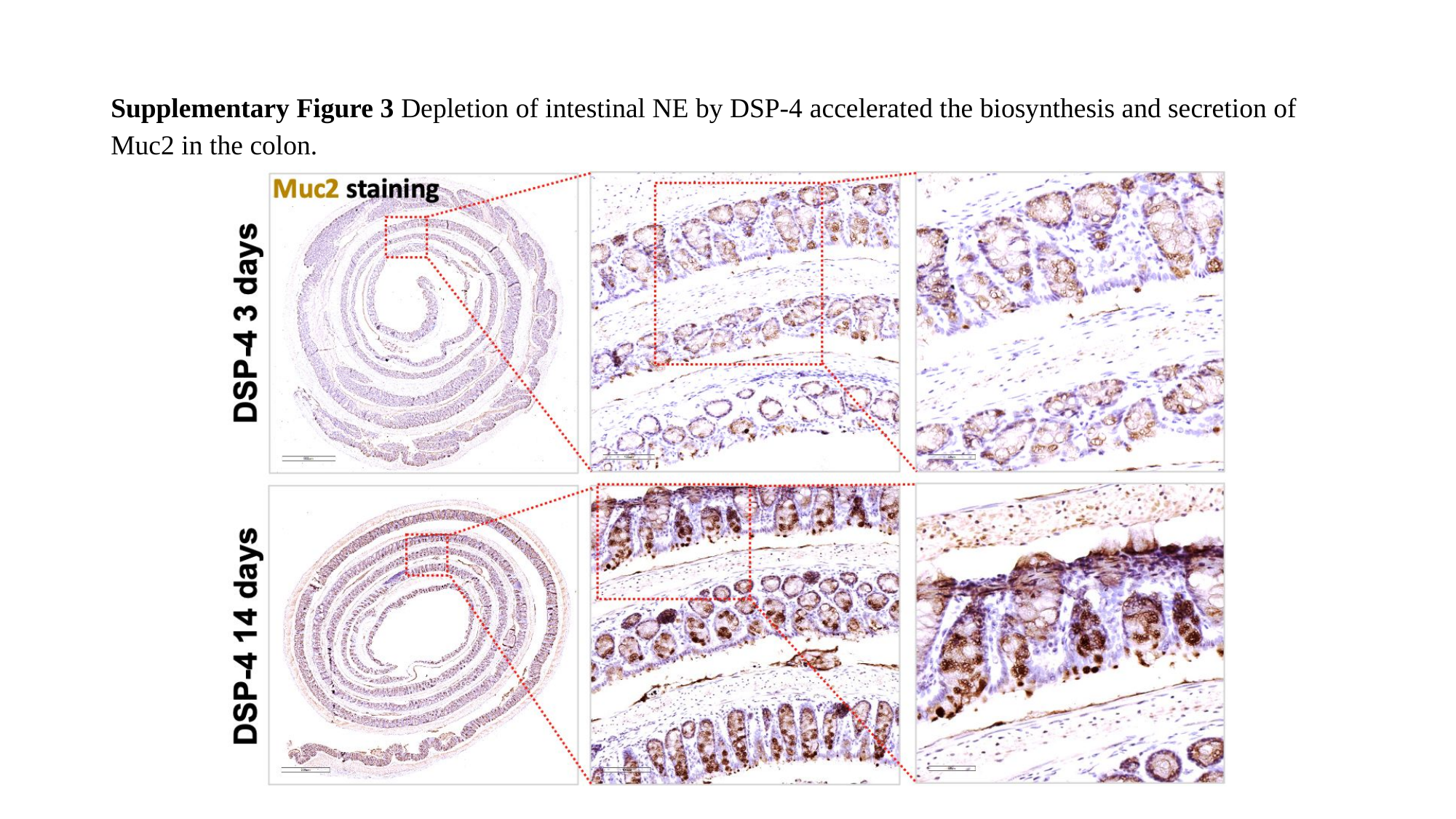

# Supplementary Figure 3 Depletion of intestinal NE by DSP-4 accelerated the biosynthesis and secretion of Muc2 in the colon.

## Slide 5
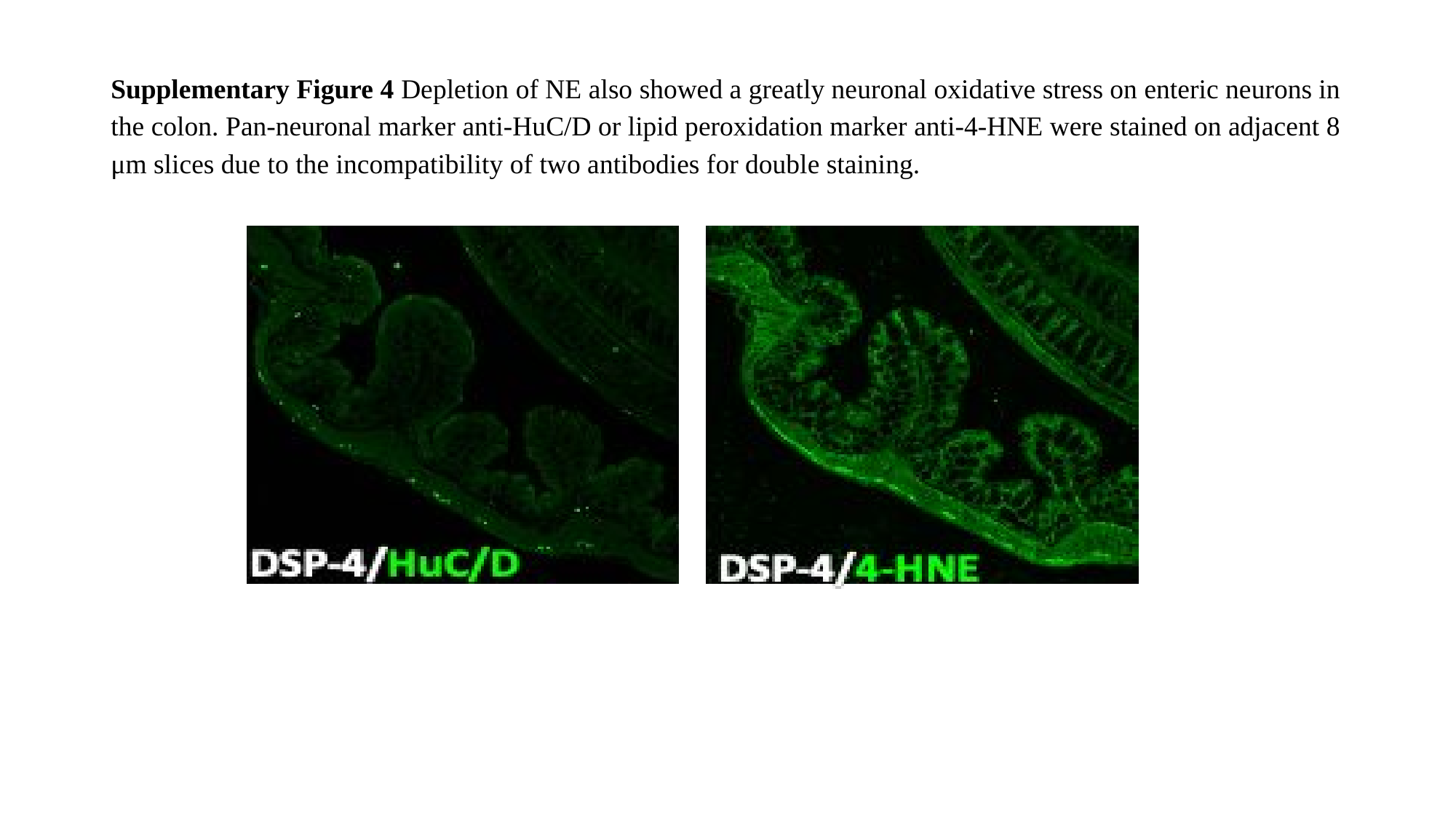

# Supplementary Figure 4 Depletion of NE also showed a greatly neuronal oxidative stress on enteric neurons in the colon. Pan-neuronal marker anti-HuC/D or lipid peroxidation marker anti-4-HNE were stained on adjacent 8 μm slices due to the incompatibility of two antibodies for double staining.

## Slide 6
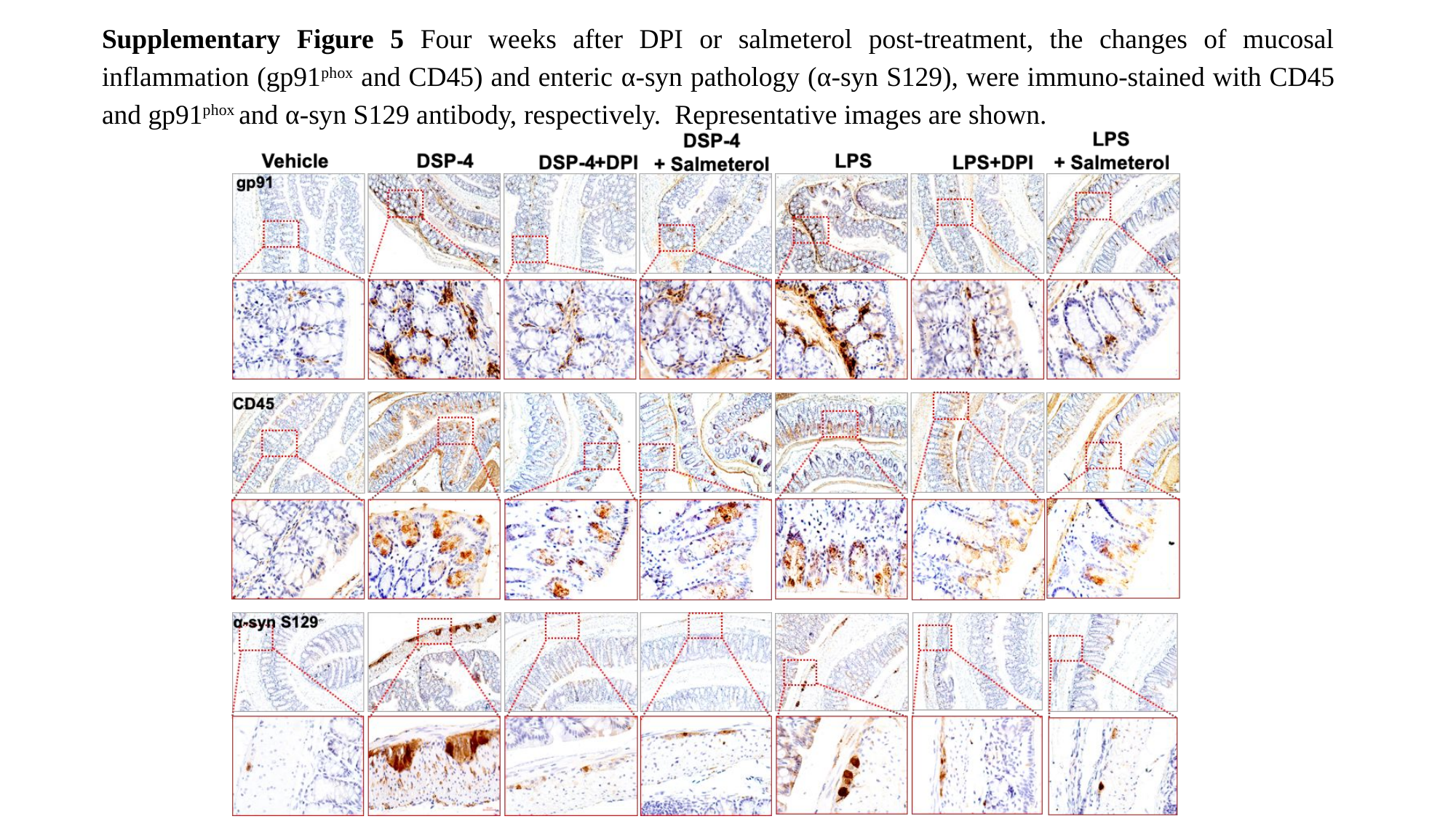

# Supplementary Figure 5 Four weeks after DPI or salmeterol post-treatment, the changes of mucosal inflammation (gp91phox and CD45) and enteric α-syn pathology (α-syn S129), were immuno-stained with CD45 and gp91phox and α-syn S129 antibody, respectively. Representative images are shown.
