# Supplementary material for: Dysfunction of the noradrenergic system drives inflammation, α-synucleinopathy, and neuronal loss in mouse colon
Source: Front Immunol. 2023 Feb 10;14:1083513. doi: 10.3389/fimmu.2023.1083513 (PMC9950510; doi:10.3389/fimmu.2023.1083513)
Supplement: Supplementary file 2 [file Presentation_2.pptx]

## Slide 1
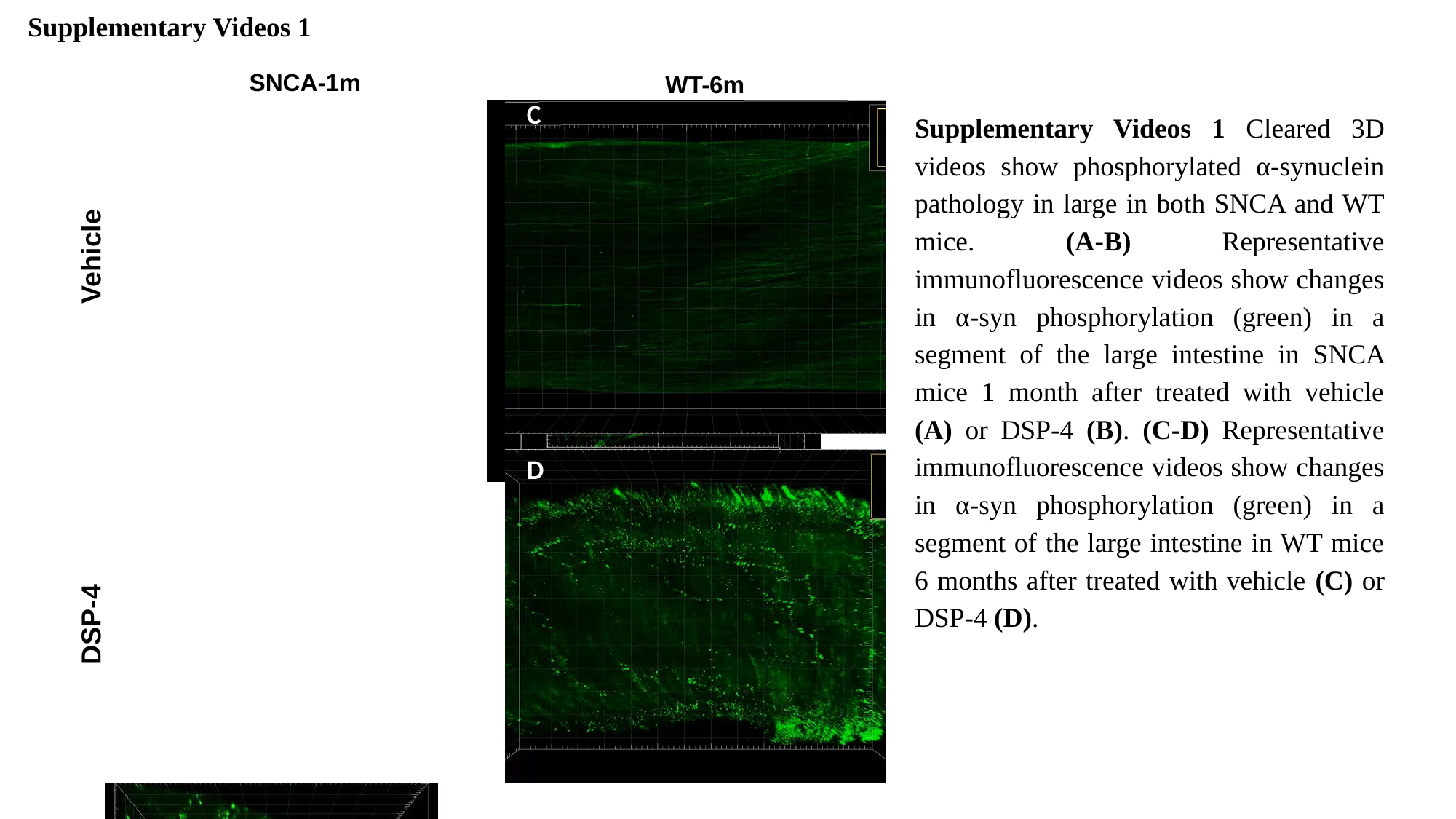

Supplementary Videos 1
SNCA-1m
WT-6m
C
A
Supplementary Videos 1 Cleared 3D videos show phosphorylated α-synuclein pathology in large in both SNCA and WT mice. (A-B) Representative immunofluorescence videos show changes in α-syn phosphorylation (green) in a segment of the large intestine in SNCA mice 1 month after treated with vehicle (A) or DSP-4 (B). (C-D) Representative immunofluorescence videos show changes in α-syn phosphorylation (green) in a segment of the large intestine in WT mice 6 months after treated with vehicle (C) or DSP-4 (D).
Vehicle
B
D
DSP-4
