# Supplementary material for: Dysfunction of the noradrenergic system drives inflammation, α-synucleinopathy, and neuronal loss in mouse colon
Source: Front Immunol. 2023 Feb 10;14:1083513. doi: 10.3389/fimmu.2023.1083513 (PMC9950510; doi:10.3389/fimmu.2023.1083513)
Supplement: Supplementary file 3 [file Presentation_3.pptx]

## Slide 1
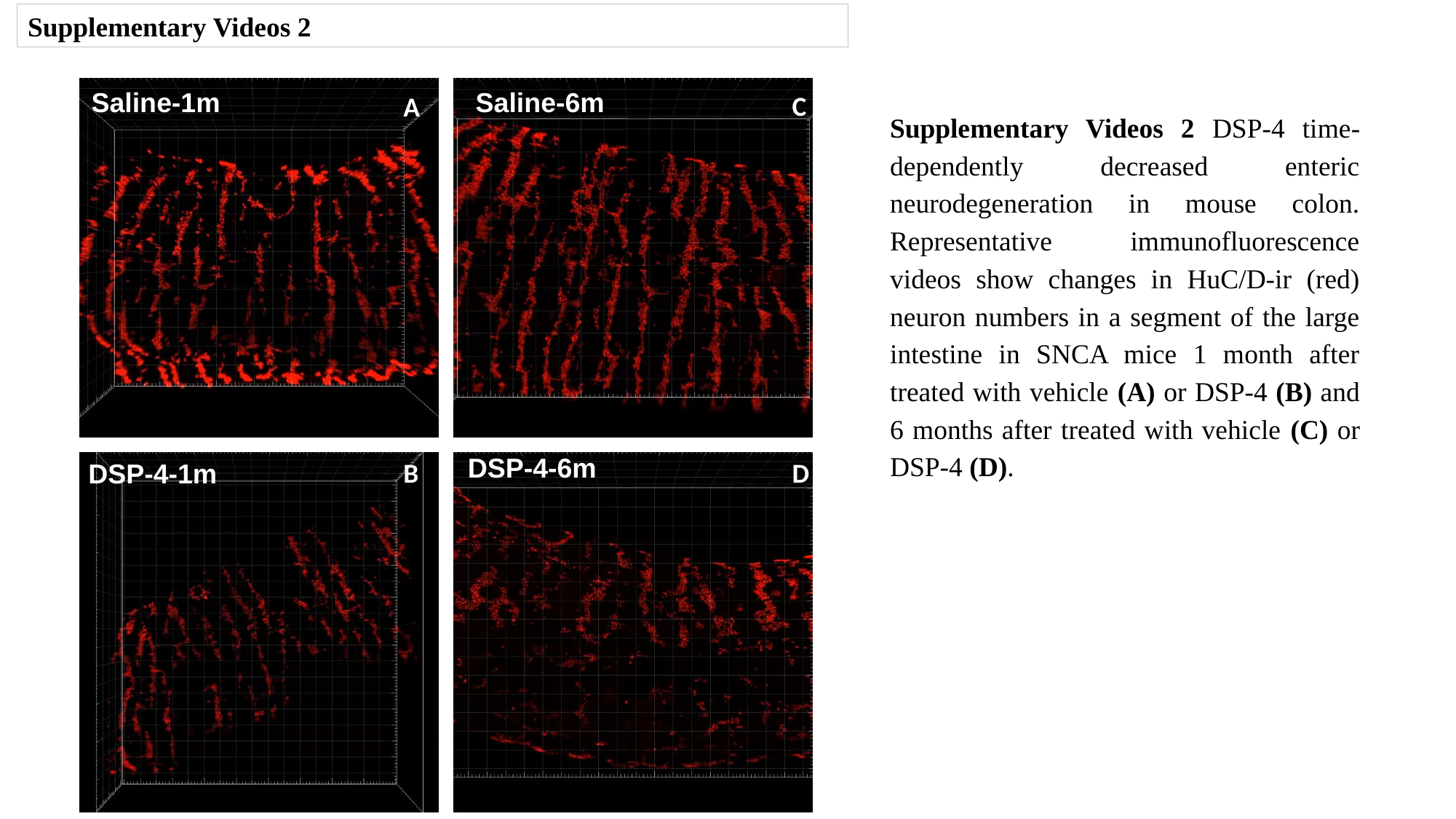

Supplementary Videos 2
Saline-1m
Saline-6m
C
A
Supplementary Videos 2 DSP-4 time-dependently decreased enteric neurodegeneration in mouse colon. Representative immunofluorescence videos show changes in HuC/D-ir (red) neuron numbers in a segment of the large intestine in SNCA mice 1 month after treated with vehicle (A) or DSP-4 (B) and 6 months after treated with vehicle (C) or DSP-4 (D).
DSP-4-6m
B
D
DSP-4-1m
