# Supplementary material for: Dysfunction of the noradrenergic system drives inflammation, α-synucleinopathy, and neuronal loss in mouse colon
Source: Front Immunol. 2023 Feb 10;14:1083513. doi: 10.3389/fimmu.2023.1083513 (PMC9950510; doi:10.3389/fimmu.2023.1083513)
Supplement: Supplementary file 4 [file Presentation_4.pptx]

## Slide 1
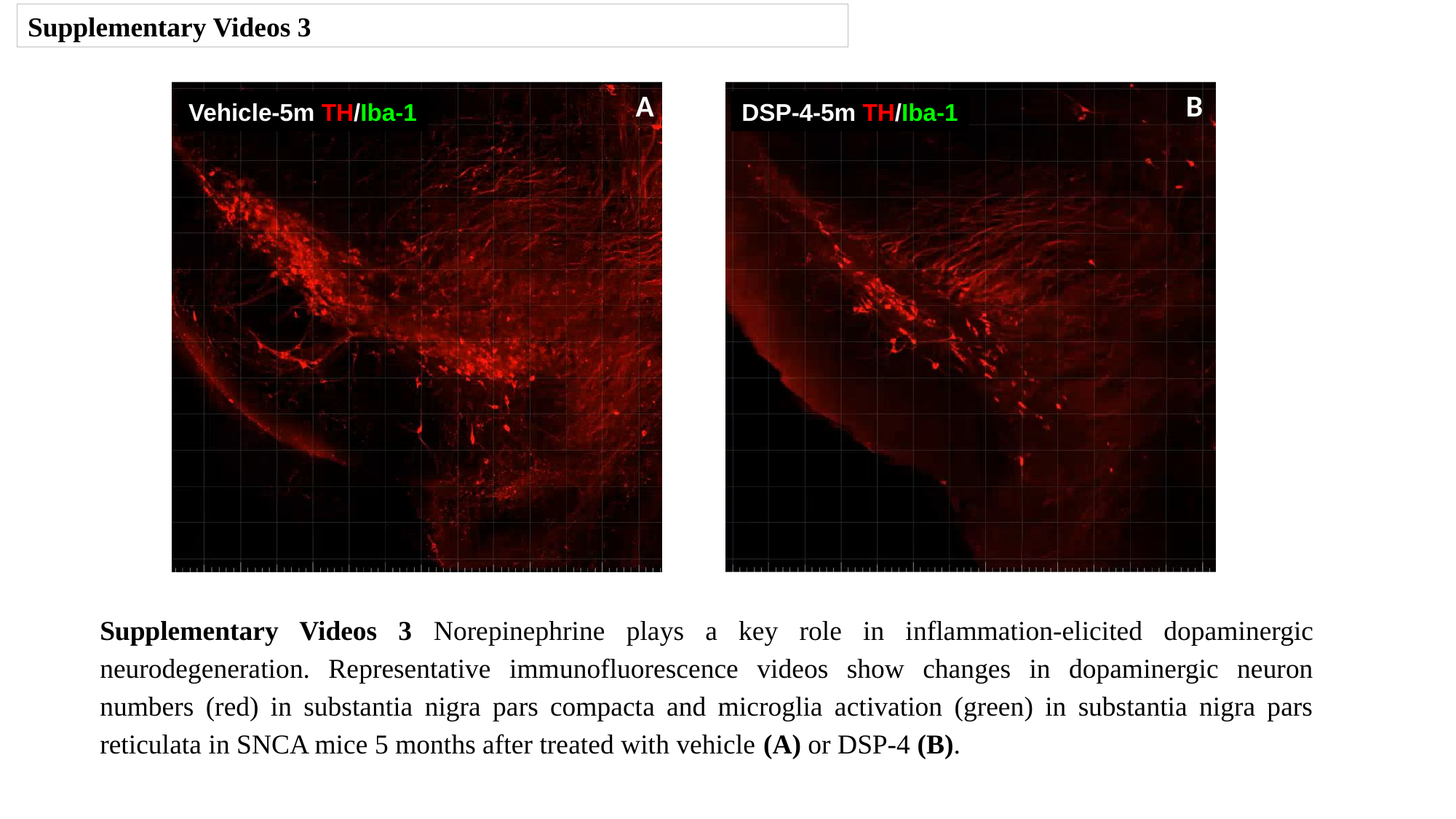

Supplementary Videos 3
A
B
Vehicle-5m TH/Iba-1
DSP-4-5m TH/Iba-1
Supplementary Videos 3 Norepinephrine plays a key role in inflammation-elicited dopaminergic neurodegeneration. Representative immunofluorescence videos show changes in dopaminergic neuron numbers (red) in substantia nigra pars compacta and microglia activation (green) in substantia nigra pars reticulata in SNCA mice 5 months after treated with vehicle (A) or DSP-4 (B).

## Slide 2
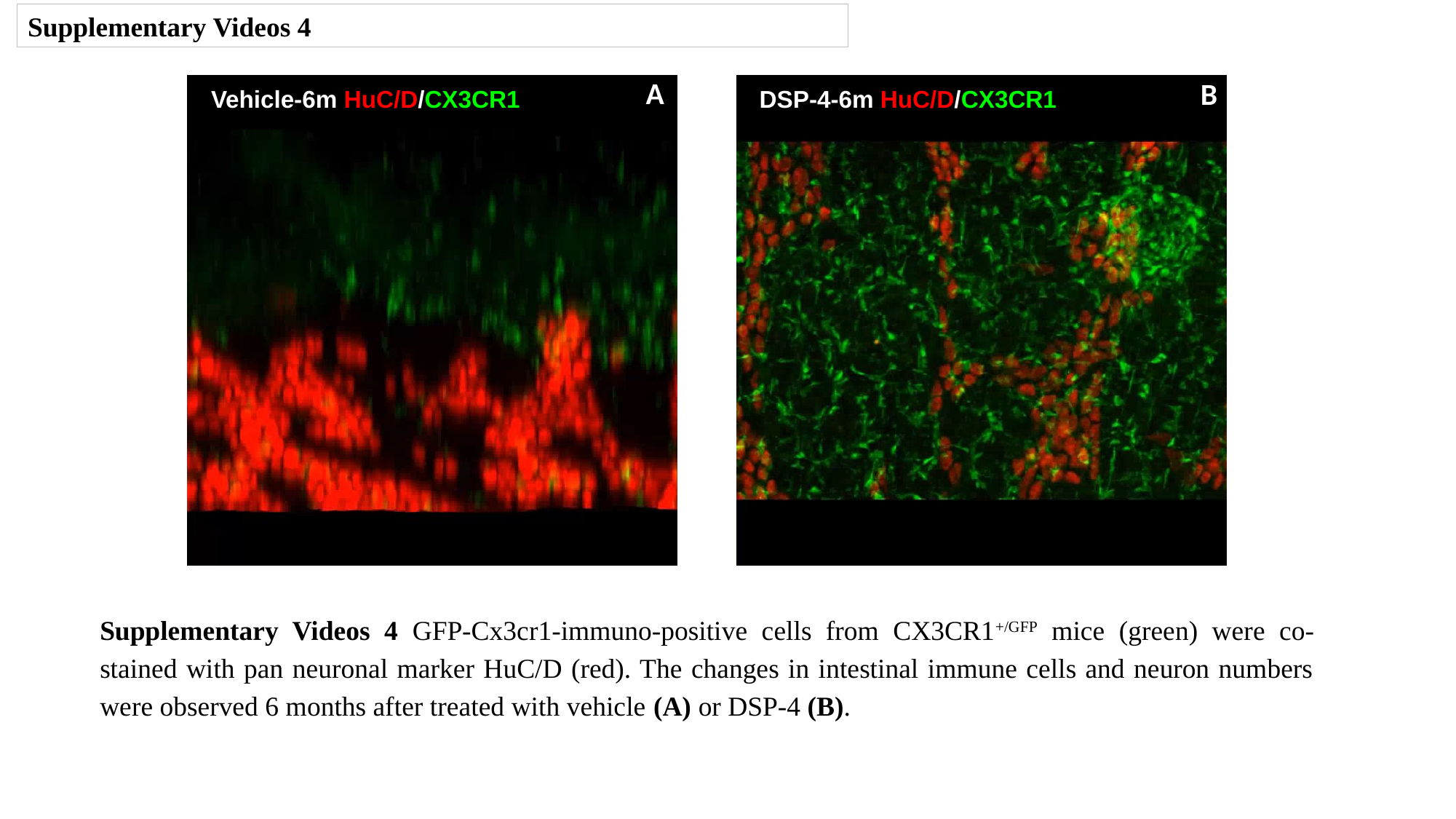

Supplementary Videos 4
A
B
Vehicle-6m HuC/D/CX3CR1
DSP-4-6m HuC/D/CX3CR1
Supplementary Videos 4 GFP-Cx3cr1-immuno-positive cells from CX3CR1+/GFP mice (green) were co-stained with pan neuronal marker HuC/D (red). The changes in intestinal immune cells and neuron numbers were observed 6 months after treated with vehicle (A) or DSP-4 (B).
